# Small-Scale Insight into Uniform Deformability and Softening Resistance of Refractory High-Entropy Alloy

**DOI:** 10.1021/acs.nanolett.5c05077

**Published:** 2026-02-19

**Authors:** Cheng-Yuan Tsai, Wen-Ju Chen, Yuan-Tao Hsu, Chi-Huan Tung, Su-Jien Lin, Jien-Wei Yeh, Shou-Yi Chang

**Affiliations:** † Department of Materials Science and Engineering, National Tsing Hua University, Hsinchu 30013, Taiwan; ‡ High Entropy Materials Center, 34881 National Tsing Hua University, Hsinchu 30013, Taiwan

**Keywords:** High-entropy alloys, lattice distortion, plasticity, dislocation activity, simulation

## Abstract

Owing to the outstanding softening resistance and thermal stability of BCC-structured refractory high-entropy alloys (HEAs), their unique deformation-induced defect structures merit investigation. Therefore, this study evaluated the mechanical properties and deformation behaviors of W-based low- to high-entropy alloys at various temperatures and orientations using nanoindentation and microcompression, complemented by post-mortem TEM and atomistic simulations to observe dislocation populations and their evolution. Results reveal that HEAs exhibit reduced elastic and plastic anisotropy while retaining high-temperature strength. With increasing compositional complexity, planar slip and abrupt stress drops were progressively replaced by homogeneous flow and smoother serrations. Severe lattice distortion promoted dislocation nucleation but impeded long-range glide, enabling cooperative edge and screw dislocation activity that sustained strength and work hardening across temperatures.

## Graphical abstract

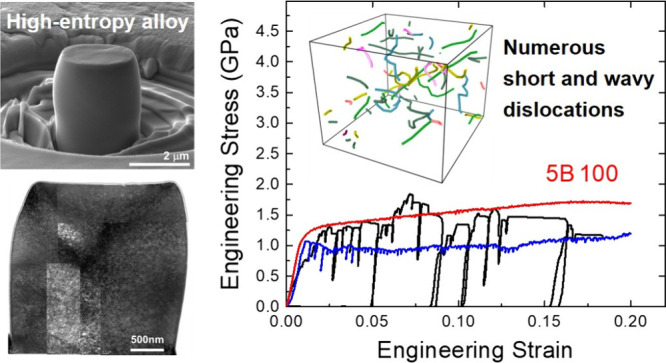

High-entropy alloys (HEAs) represent a paradigm shift in alloy design. Unlike conventional alloys based on a single principal element, HEAs are composed of multiple elements in near-equiatomic ratios, forming stable solid-solution phases.^1^ This approach enables unprecedented compositional flexibility, opening new avenues in materials research.^2,3^ HEAs are characterized by four core effects, including high-entropy, sluggish diffusion, severe lattice distortion, and cocktail effects,^1,4–10^ which endow them with a wide range of distinct mechanical performances. For instance, FCC-structured CoCrFeMnNi HEAs activate multiple twinning systems at low-temperatures, enhancing strain hardening and plasticity.^11–15^ Similarly, BCC-structured WTaMoNb micropillars exhibit negligible grain boundary migration and grain growth during deformation at 600 °C due to sluggish diffusion.^16^

Recent studies have shown that BCC-structured HEAs maintain phase stability and exhibit exceptional softening resistance at elevated temperatures. For example, WTaMoNbV HEAs retain a yield strength of 477 MPa at 1600 °C.^17^ The outstanding performance arises from the combined effects of dominant edge dislocation strengthening and pronounced solid-solution strengthening. In addition, Curtin et al. established a simulated model incorporating edge dislocations as the primary strengthening mechanism, successfully predicting yield strength that closely matched experimental values for MoNbTaW and MoNbTaVW HEAs. These results suggest that edge dislocations may play a more significant role in refractory HEAs than previously expected.^18^ SEM observations after compression tests at room temperature and 1173 K also revealed that NbTaTiV and CrMoNbV HEAs retained abundant {110}⟨111⟩ edge dislocations, providing evidence of active edge dislocation mechanisms during plastic deformation.^19^

In recent years, efforts have focused on correlating the mechanical performance of HEAs with underlying deformation mechanisms, such as lattice distortion, which stems from variations in lattice constants, bonding energies, atomic size differences, and mutual interactions.^2,7,20–24^ Ma et al. utilized atomistic simulations to reveal that distortions generated a rough energy landscape, resulting in rugged and twisting dislocation loops, as well as reduced edge dislocation mobility approaching that of screw dislocations.^18,25,26^ Besides, lattice distortion reduces stacking fault energy, facilitating dynamic recrystallization as observed in HfNbTaTiZr HEAs during high-temperature deformation, where fine grains form near grain boundaries to retain strength.^19,26–28^ For highly distorted TaNbHfZrTi HEAs, Fu et al. replaced Ta with Mo, which has a larger atomic size mismatch, forming BCC-structured MoNbHfZrTi HEAs. The resulting yield strength was significantly enhanced across temperatures, indicating increased lattice distortion enhances solution strengthening.^29,30^ However, the effect of lattice distortion on the deformation behaviors of WTaMoNbV HEAs under various conditions remains unclear and worth systematic investigation.

Therefore, this study investigates the orientation-dependent mechanical properties of BCC HEAs to elucidate the effect of lattice distortion. Observations of the effects of multielement addition and temperature on crystallographic anisotropy and defect evolution are also required. To address these questions, nanoindentation is employed to measure the Young’s modulus and hardness of BCC-structured pure tungsten, medium-entropy alloys (MEAs), and high-entropy alloys (HEAs) across temperatures and orientations. The mechanical properties and elastic-plastic anisotropy influenced by lattice distortion and temperature are systematically examined. Furthermore, microcompression tests and the subsequent cross-sectional analyses of deformed pillars are used to investigate the correlation between lattice distortion and defect evolution during plastic deformation. Molecular dynamics (MD) simulations are also implemented to observe dislocation morphology and quantify dislocation structures, offering insights into the temperature-dependent mechanical properties and deformation mechanisms in HEAs.

## Microstructure and Mechanical Properties

Figure 1 presents the EBSD IPF maps and XRD patterns of the 1B–5B alloys, along with the averaged values and cumulative plots of Young’s modulus and hardness for the 1B, 3B and 5B alloys along ⟨100⟩ and ⟨111⟩ stress orientations at different temperatures (Figure S1 in the Supporting Information). EBSD results reveal equiaxed, randomly oriented grains across all alloys. Due to rapid grain growth during homogenization, 1B exhibits the coarsest grains, whereas 4B and 5B display much finer microstructures (∼100 μm), attributed to sluggish diffusion in multiprincipal systems.^8^ XRD confirms that all alloys retain a single-phase BCC structure. The atomic size difference (δ) and the parameter of lattice distortion (δ_l_), both summarized in Table S2 in the Supporting Information, provide quantitative measures of lattice distortion from both the predicted and experimentally observed perspectives. These parameters generally increase with the number of constituent elements, with 5B exhibiting the most pronounced lattice distortion among all alloys. Figure 1C further compares experimental (a_Avg._) and theoretical lattice constants (a_Theory_), where 5B shows the largest deviation (0.22%), in agreement with the parameters quantified earlier and reinforcing that 5B exhibits the most severe lattice distortion arising from its chemical complexity (Table S3 in the Supporting Information).

**Figure 1:**
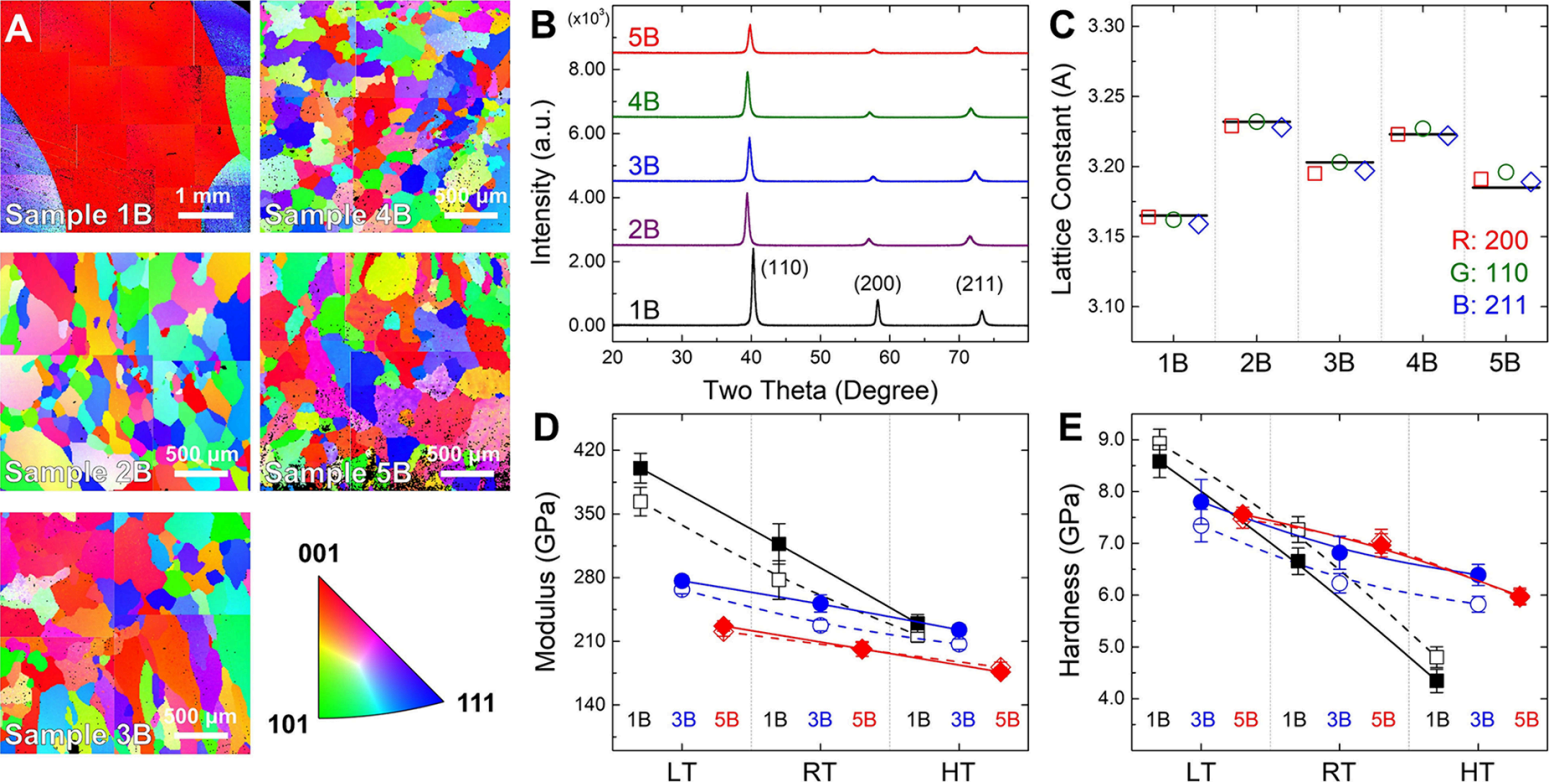
1B–5B alloys: (A) EBSD IPF (grain orientation) maps, (B) XRD patterns, (C) experimentally determined lattice constants from XRD peak analysis (black line: a_Theory_); temperature-dependent averaged values of the nanoindentation results for the 1B, 3B and 5B alloys: (D) Young’s modulus and (E) hardness (solid: ⟨100⟩, open: ⟨111⟩ stress orientation).

To further elucidate the effect of lattice distortion on mechanical behavior, nanoindentation reveals orientation-dependent elastic and plastic responses, as shown in Figure 1D,E, with the corresponding elastic and plastic anisotropy indices and anisotropy reduction ratios summarized in Table S4 in the Supporting Information. Regarding Young’s modulus, all alloys exhibit higher values along ⟨100⟩, with A_E_ increasing from 0.875 (1B) to 0.904 (3B) and approaching unity in 5B (0.997) at RT. This corresponds to elastic anisotropy reduction ratios R of 23.0% and 97.4% for 3B and 5B, respectively. With increasing temperature, 1B experiences a steep modulus drop, whereas 3B and 5B remain relatively stable due to distortion-mediated thermal resistance.^31^ Notably, 5B persistently maintains high R values of 70.2% (A_E_ = 0.973) at LT and 153.5% (A_E_ = 1.030) at HT, significantly outperforming 3B (R ≈ 23–62%) and the baseline 1B. This robust, thermally stable elastic isotropy is closely associated with the peak lattice distortion in 5B (δ = 3.617% and δ_l_ = 1.31%), which generates large static atomic displacements analogous to thermal vibrations.^32^ It confers strong resistance to thermal perturbations and redistributes atomic bonding forces,^33^ thereby minimizing directional stiffness differences. In terms of hardness, 1B shows strong plastic anisotropy (A_H_ = 1.092) at RT, with higher hardness along ⟨111⟩. In contrast, 3B displays a reversed trend (A_H_ = 0.914) driven by orientation-dependent slip evolution,^34^ yet yields only a marginal anisotropy reduction of 6.0%. Notably, 5B approaches near-isotropic plasticity (A_H_ = 1.012), corresponding to a substantial anisotropy reduction of 86.6%. This abrupt and disproportionate suppression of plastic anisotropy, with R jumping from 6.0% to 86.6%, indicates a distortion-mediated threshold effect in slip activation as δ_l_ increases from 1.29% to 1.31%, rather than a gradual homogenization of bonding stiffness. As indicated by Gianola et al., such high distortion levels in refractory multiprincipal element alloys equalized slip resistance across slip planes, facilitating the activation of rarely observed higher-order slip systems and promoting more uniform plastic deformation.^35^ At elevated temperatures, 1B softens significantly, consistent with the behavior of pure tungsten,^36,37^ whereas 3B and 5B retain high hardness due to strong solid-solution strengthening and enhanced lattice friction from distortion. Specifically, severe lattice distortion substantially increases the Peierls barrier, restricting long-range dislocation glide and suppressing localized deformation under elevated temperatures,^18,25,26,38^ as reflected by the still significant anisotropy reduction ratio of 105.5% in 5B at HT.

## Microcompression and Plastic Deformation Behaviors

Figure 2 shows the stress–strain curves along with corresponding serration statistics and cumulative plots for the 1B, 3B and 5B ⟨100⟩ micropillars at various temperatures (Figure S2 in the Supporting Information). First, the evolution of yield strength demonstrated in Figure 2D from 1B to 5B alloys across temperatures can be interpreted using the solid-solution strengthening model.^26^ Based on this model, the critical resolved shear stress for dislocation glide is expressed as $\tau_{y}\left(T,\mathrm{\varepsilon ̇}\right)=\tau_{y0}\exp\left[-\left(1/0.55\right){\left(\left(\mathrm{kT}/\Delta E_{b}\right)\ln\left(\overset{˙}{\varepsilon_{0}}/\mathrm{\varepsilon ̇}\right)\right)}^{0.91}\right]$ , where ε̇ is plastic strain rate, ε̇_0_ = 10^4^s^–1^;^39^ the 0 K flow stress τ_y0_ and energy barrier ΔE_b_ are given by $\tau_{y0}=1.01{\left(\Delta {Ẽ}_{p}^{4}\left(w_{c}\right)/\Gamma b^{5}w_{c}^{5}\right)}^{1/3}$ , $\Delta E_{b}=1.11{\left(w_{c}^{2}\Gamma \Delta {Ẽ}_{p}^{2}\left(w_{c}\right)/b\right)}^{1/3}$ . Here, ΔẼ_p_ (w) stands for the energy variation per unit dislocation length due to local potential fluctuations. The progressively reduced temperature sensitivity from 1B to 5B arises from larger ΔẼ_p_(w) and ΔE_b_ associated with increasing chemical complexity. Notably, 5B exhibits the highest yield strength retention at elevated temperatures, implying the roughest energy landscape and most resistant dislocation glide. As a result, pronounced hardening is achieved in the fully aligned 5B100-2 micropillar due to abundant and strong dislocation interactions, as shown in Figure 2B.

**Figure 2:**
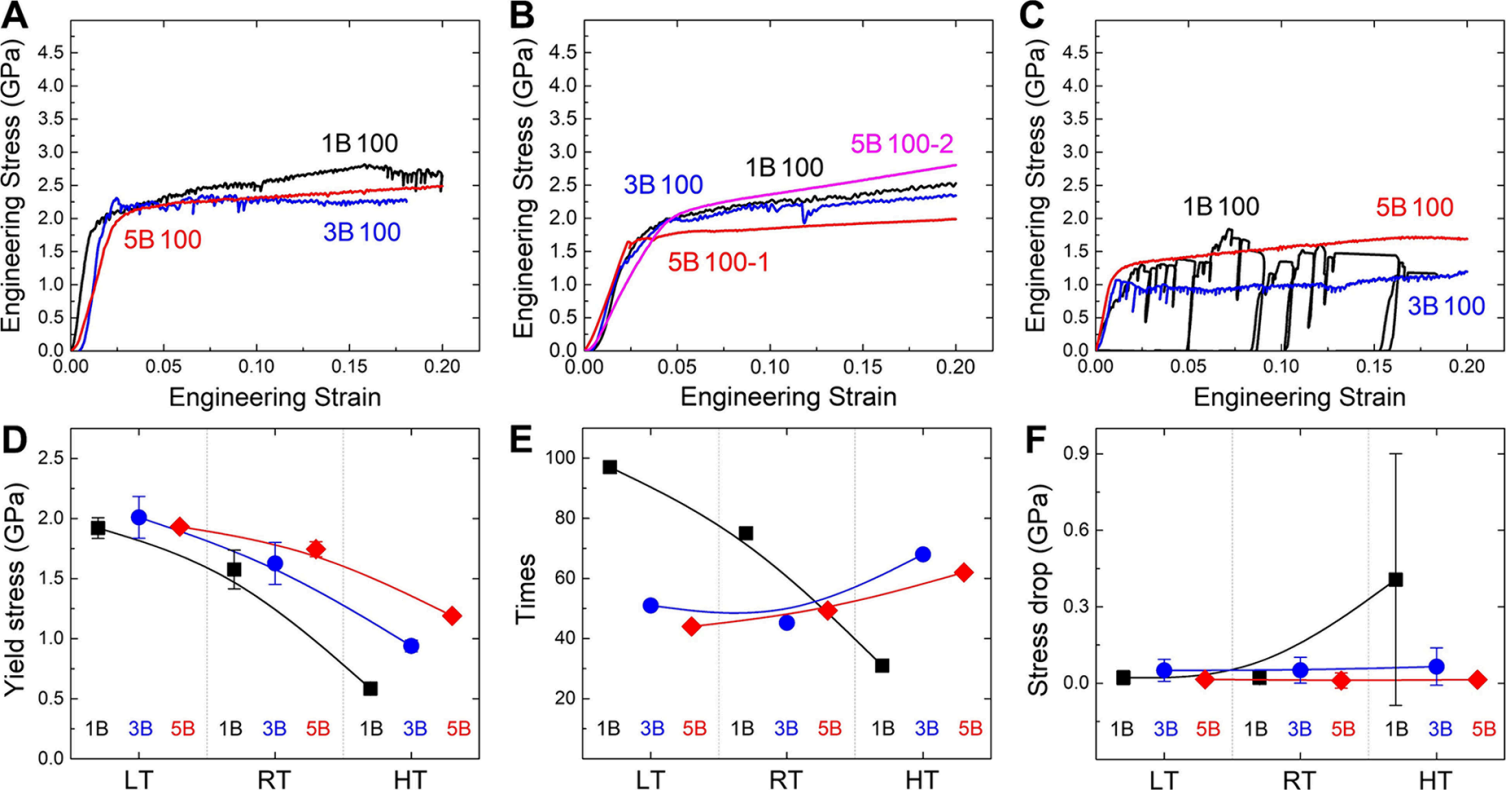
Representative compressive stress–strain curves of the 1B, 3B and 5B ⟨100⟩ micropillars at different temperatures: (A) LT, (B) RT, and (C) HT; (D) yield stress; statistical analysis of serration behavior during plastic deformation: (E) frequency and (F) magnitude of stress drops. For each alloy, four micropillars were tested at RT, and three micropillars at LT and HT.

Although 5B displays the best high-temperature performance among the three alloys, its performance should be further contextualized through systematic comparison with other state-of-the-art refractory HEAs and conventional superalloys commonly used in high-temperature applications, in order to assess its practical applicability at elevated temperatures (Table S5 in the Supporting Information). It is worth noting that severely distorted 5B typically exhibits a weak size effect on strength,^40^ rendering the mechanical strength measured in this study comparable to bulk results reported in the literature. Specifically, 5B exhibits a yield strength of 1193 MPa at 300 °C, while its strength retention ratio (0.684) is lower than that of superalloys such as Mar-M247 and Inconel 718 at comparable temperatures.^73,74^ Nevertheless, its absolute yield strength remains superior to the peak strength of single-crystal CMSX-4 superalloys^75^ (∼1120 MPa at 750 °C, the highest value among the three superalloys considered), providing a significant safety margin. Crucially, the full potential of 5B lies in the extreme temperature regime (>1000 °C). For instance, the polycrystalline MoNbTaVW alloy (same nominal composition as 5B) retains a yield strength of 656 MPa with a retention ratio of 0.526 even at 1400 °C.^17^ Such thermal stability contrasts sharply with other classes of single-phase refractory HEAs at ultrahigh temperatures: Hf-containing alloys undergo rapid softening^76^ (e.g., HfNbTaTiZr drops to 92 MPa at 1200 °C,^77^ while ⟨111⟩ single-crystal HfTaTiVZr shows an approximately 50% strength reduction already at 800 °C);^78^ Al-containing alloys often lose strength due to phase transformations;^79^ and V/Cr-rich lightweight alloys exhibit limited strength retention above 800 °C.^80^ Accordingly, based on systematic comparisons with commonly used candidate materials, the 5B alloy, particularly in the single-crystal form without grain boundary sliding, suggests strong potential for high-temperature micromechanical applications.

To further probe the deformation dynamics, Figure 2E,F display statistical analyses of stress drop frequency and magnitude, revealing qualitatively consistent and reproducible serration behavior across repeated tests for each testing condition. In general, recent studies indicate that stress drops originate primarily from dislocation avalanches, characterized by intermittent collective dislocation motion and sudden elastic energy release as dislocation bursts approach the surface, generating single or multiple slip bands.^41–43^ As temperature increases, 1B exhibits fewer but larger stress drops, primarily due to enhanced screw dislocation mobility via thermally activated kink-pair formation and glide at lower stress levels.^44,45^ In contrast, the number of stress drops in 3B and 5B rises with temperature. Studies have shown that elevated temperatures lower defect formation barriers, while the complex chemical environment further promotes localized dislocation nucleation, as seen in Mo-rich regions of WTaMoNb HEAs where weak bonding favors local stress release.^46,47^ Similarly, more activation of the distortion-induced soft regions intrinsically present in multicomponent 3B and 5B would trigger more frequent short-range dislocation nucleation and subsequent dislocation avalanches with rising temperature, thereby producing more stress drops, in contrast to the descending trend in the chemically homogeneous 1B. However, rough energy landscape in HEAs limits strain transfer by trapping dislocations,^48^ leading to intermittent motion on multiple slip systems^49^ and resulting in smaller individual slip events and stress drops across temperatures.

Figure 3A–C present the postcompression SEM micrographs of the 1B, 3B, and 5B ⟨100⟩ micropillars (#1) deformed at RT. Additional representative micropillars (#2 to #4) are shown in Figure S3 in the Supporting Information, with real-time deformation behavior captured through *in situ* SEM videos (Videos S1–S12 in the Supporting Information). Postcompression SEM micrographs of the 1B, 3B, and 5B ⟨100⟩ micropillars deformed at LT and HT are displayed in Figure S4 in the Supporting Information. It is shown that slip traces are unclear in 1B at low temperatures but appear wavy due to sluggish screw dislocation motion and active cross-slip. With increasing temperature, the higher T_test_ /*T*_c_ ratio enhances screw dislocation mobility, suppresses cross-slip, and activates distinct slip systems,^45^ consistent with findings in pure W micropillars.^50^ In 3B, the addition of Ta and Mo lowers *T*_c_, resulting in a higher T_test_ /*T*_c_ ratio and clearer slip traces. Although adding Nb and V to form 5B further increases T_test_ /*T*_c_ ratio, the micropillars instead exhibit isotropic expansion with only subtle slip traces except at HT. This is attributed to spontaneous kink formation in screw dislocations, allowing cross-slip on multiple planes and move along various directions to accommodate local solute environments.^51,52^ Besides, high temperatures (HT) facilitate the identification of activated slip systems through analysis of slip trace angles relative to the loading direction and corresponding Schmid factors (Table S6 in the Supporting Information). For 1B, the measured angle of 56.6° closely matches the maximum Schmid factor for the (211)­[111] slip system. In 3B, angles ranging from 53° to 54° across temperatures also imply the activation of (211)­[111] and (312)­[111] slip systems. In contrast, the 44.4° angle in 5B matches the (110)­[111] slip system, which has low resolved shear stress but lower slip resistance for dislocation glide.^53^ This suggests that complex chemical environments and severely distorted structure alter slip resistance, indicating that the activation of slip systems depends on both resolved shear stress and slip resistance.

**Figure 3:**
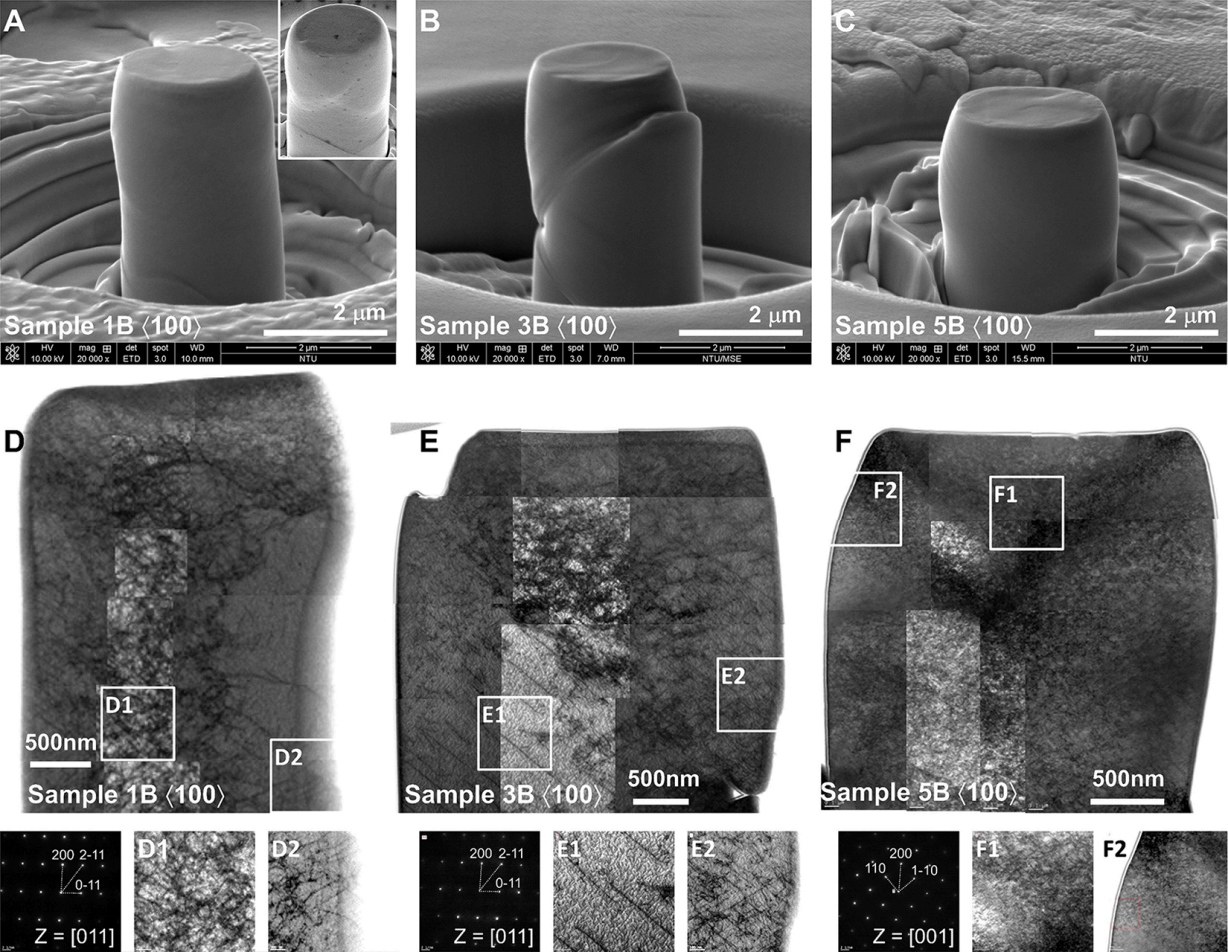
Postcompression SEM micrographs and corresponding cross-sectional STEM images, including selected-area diffraction patterns and magnified views of deformation zones 1 and 2, for ⟨100⟩ micropillars (#1) deformed at RT: (A and D) 1B (inset: different viewing angle), (B and E) 3B, and (C and F) 5B.

## Deformed Micropillar Defect Structures and Activities

Figure 3D–F present the cross-sectional STEM images of 1B, 3B, and 5B ⟨100⟩ micropillars (#1) deformed at RT. Magnified views of deformation zones 1 and 2, accompanied by schematic projections and slip vectors, are provided in Figure S5 in the Supporting Information. In 1B, two types of 1/2⟨111⟩ dislocations are activated, with significant entanglement from active screw dislocation cross-slip.^45,50^ Straight slip lines near the surface indicate long-range slip along specific systems. In 3B, deformation is dominated by dislocation entanglement in the upper region and planar slip in the lower part. Despite higher slip resistance, slip plane activation remains localized. In 5B, dense curved dislocations and fine loops indicate easy defect formation but limited growth.^47^ Severe lattice distortion reduces slip resistance differences,^35^ promoting activation of multiple slip systems. The rough energy landscape further raises the Peierls barrier,^25^ restricting dislocation motion and suppressing surface slip bands. To further examine the temperature effect on defect evolution in HEAs, Figures S6 and S7 in the Supporting Information demonstrate cross-sectional STEM images and statistical analyses of dislocation character for 5B ⟨100⟩ micropillars deformed at different temperatures. As temperature increases, cross-kink annihilation becomes more prominent, slightly reducing screw dislocation density.^54,55^ In contrast, edge dislocations remain strongly trapped,^56^ limiting their motion to the surface even at high temperatures. Notably, quantitative analysis reveals that edge dislocations (26–30 counts) consistently outnumber screw dislocations (17–22 counts) across all testing temperatures. This behavior contrasts with the screw dislocation-dominated plasticity typically observed in conventional BCC metals^57^ and suggests a distortion-induced shift in the dominant deformation mechanism. The relatively high density of randomly distributed edge segments with multiple slip vectors promotes frequent dislocation interactions and entanglements. These entangled substructures serve as effective barriers that counteract the thermal softening typically observed during single-slip deformation at high temperatures,^58^ thereby suppressing strain localization and promoting more uniform deformation. Furthermore, pronounced dislocation interactions significantly restrict dislocation motion and enhance the likelihood of dislocation multiplication. This mechanism facilitates the work hardening evidenced by the monotonic increase in flow stress observed for 5B, as shown in Figure 2A–C. Moreover, the dense stress fields in 5B could significantly hinder dislocation expansion.^25^ Therefore, fine loops and entangled dislocation clusters are consistently observed, while long and straight dislocation lines are rarely seen.

Figure 4A–C display the magnified cross-sectional views of the top regions of deformed 1B and 5B ⟨100⟩ micropillars at RT and HT. In 1B, deformation is dominated by long and straight screw dislocations gliding over long distances, primarily along a single direction on parallel slip systems. In 5B, short dislocations are densely and uniformly distributed at both room and high temperatures, gliding over short distances in multiple directions with strong interactions. Edge and screw dislocations are present in comparable amounts, collectively governing plastic deformation. However, chemical complexity affects the energy barriers (ΔE_b_) and Peierls stresses of these two dislocation characters in distinct ways. For screw dislocations, Yin et al. reported that fluctuations in the equilibrium core energies of dislocation dipoles in MoNbTaW refractory HEAs produce a broad distribution of ΔE_b_, spanning approximately −0.6 to +0.7 eV/b, far exceeding the narrow range of their constituent elements (∼0.03–0.09 eV/b).^54^ This hierarchical energy landscape imposes strong trapping effects, markedly increasing the effective Peierls stresses required for screw dislocation motion.^52^ In contrast, for edge dislocations, their intrinsic ΔE_b_ is less sensitive to local chemical fluctuations because of the planar core structure. Instead, chemical complexity amplifies solute-dislocation interaction energies, leading to a much higher extrinsic ΔE_b_ and the corresponding friction stress, which in turn enhances high-temperature strength retention.^26^ Figure 4D–F show the lattice images and strain field of 5B ⟨100⟩ micropillars deformed at RT, while the complete results across all temperatures are provided in Figures S8–S10 in the Supporting Information. Dislocation analysis reveals that Burgers vectors belong to the 1/2⟨111⟩ family $\overset{⇀}{b_{1}}=1/2\left[11\mathrm{1̅}\right]$ , $\overset{⇀}{b_{2}}=1/2\left[111\right]$ , $\overset{⇀}{b_{3}}=1/2\left[1\mathrm{1̅}1\right]$ , $\overset{⇀}{b_{4}}=1/2\left[\mathrm{1̅}11\right]$ ). In BCC pure metals, when dislocations from different 1/2⟨111⟩ directions intersect, ⟨100⟩ dislocations ( $\overset{⇀}{b_{5}}$ ) may form to reduce dislocation line energy ( $-\overset{⇀}{b_{1}}+\overset{⇀}{b_{2}}\to \overset{⇀}{b_{5}}$ or $\overset{⇀}{b_{3}}+\overset{⇀}{b_{4}}\to \overset{⇀}{b_{5}}$ ).^59,60^ In contrast, Alhafez et al. reported that 1/2⟨111⟩ dislocations possess the lowest energy in the HfNbTaTiZr refractory HEA, with ⟨100⟩ dislocations appearing only under intense stress fields.^61^ Similarly, Yin et al. showed that ⟨100⟩ dislocation junctions are intrinsically unstable in TiZrNb HEAs due to strong local lattice distortion.^62^ Despite their instability, the activation of multiple {110} slip planes in 5B substantially increases the probability of dislocation interactions, promoting frequent formation of multijunctions that transiently generate ⟨100⟩ segments, analogous to the ⟨100⟩ binary junctions observed in TEM studies of biaxial-rolled HfNbTiZr HEAs.^62^ Once formed, these transient ⟨100⟩ segments encounter locally elevated barriers in 5B, postponing their annihilation and serving as effective pinning points. Besides, ⟨100⟩ dislocations lack suitable slip planes, making them immobile and pinning the intersecting 1/2⟨111⟩ dislocations at both ends. This reduces overall slip velocity but promotes dislocation multiplication and work hardening. Besides, lattice image analysis reveals dense Burgers circuits in multiple directions within the complex strain field, severely restricting dislocation motion.^33,63^ With increasing temperature, 1/2⟨111⟩ dislocations initially decrease and then increase, while ⟨100⟩ dislocations decline consistently. At LT, limited dislocation mobility hinders surface annihilation, increasing the possibility of 1/2⟨111⟩ dislocations forming ⟨100⟩ dislocations, which in turn intensify interactions and further impede dislocation glide. At RT, increased dislocation mobility promotes surface annihilation, but robust dislocation interactions still sustain a measurable population of ⟨100⟩ dislocations. At HT, dislocation mobility remains limited by the complex chemical environment,^54^ resulting in a relatively high dislocation density. However, dislocations primarily propagate on specific slip planes, which reduces cross-plane interactions and leads to fewer ⟨100⟩ dislocations.

**Figure 4:**
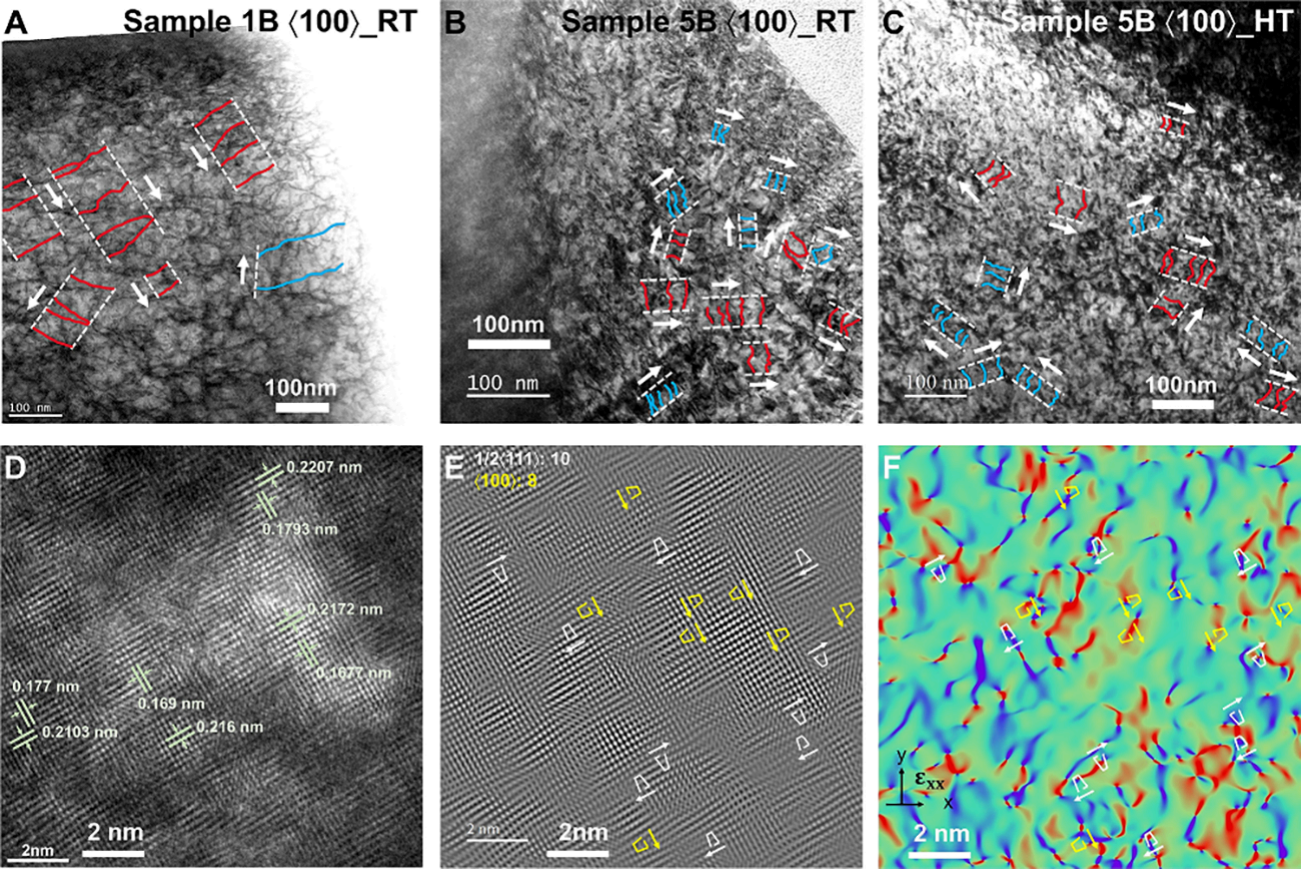
Magnified cross-sectional views of the top regions of ⟨100⟩ micropillars deformed at different temperatures: (A) 1B_RT, (B) 5B_RT, and (C) 5B_HT (red/blue solid lines: screw/edge dislocation; white dashed line: dislocation slip trace; white arrow: dislocation slip direction); lattice images of deformation zone 1 in the 5B ⟨100⟩ micropillar deformed at RT: (D) atomic-resolution, (E) filtered iFFT, and (F) strain field (blue: compressive; red: tensile) with Burgers circuits (arrow: slip direction; numbers: counts of 1/2⟨111⟩ and ⟨001⟩ dislocations).

## Dislocation Activities at Atomic Scale: Atomistic Simulation

Simulated stress–strain curves and dislocation evolution along ⟨100⟩ loading direction (Figures S11 and S12 in the Supporting Information) reveal distinct behaviors between 1B and 5B. To quantify these differences and assess the effect of chemical complexity, the averaged yield strain across all temperatures increases from 8.27% in 1B to 10.83% in 5B (5*B*/1B ratio = 1.31), closely matching the experimental compressive yield-strain ratio of 1.37 (1B: 1.46%; 5B: 2.00%) presented in Figure 2. It is noted that MD simulations exhibit much larger absolute yield strains than experiments due to the extremely high strain rates and initial defect-free structures. The ultrahigh strain rate suppresses thermally activated dislocation processes by limiting reaction time,^64^ while the absence of pre-existing defects in the simulation cell requires stresses approaching the theoretical shear strength for dislocation nucleation.^64,65^ Despite these differences, the consistency of the yield-strain ratio indicates that, although dislocation nucleation occurs earlier in 5B, severe lattice distortion strongly impedes their subsequent glide and multiplication, resulting in a delayed onset of yielding. Concerning temperature effects, in 1B, increasing temperature leads to reduced yield strength and sharp stress drops, associated with long-range dislocation glide. Although 1B retains long and straight dislocations at all temperatures, rising temperature lowers the critical shear stress,^66,67^ making dislocations more mobile and prone to escape the crystal, with significant variations in their morphology and density at high strain. In contrast, 5B exhibits a gradual elastic–plastic transition, attributed to severe lattice distortion and a rough energy landscape that lowers and spatially distributes nucleation barriers,^68^ facilitating random dislocation formation throughout the crystal. At RT, deformation is governed by dense, curved dislocations undergoing localized twisting and short-range motion along multiple directions. Compared to 1B, 5B shows reduced temperature sensitivity, reflected in smaller yield strength variations and minimal stress drops. At both LT and HT, dislocation activity is characterized by the continuous formation and slow expansion of fine dislocation loops, consistent with experimental observations.

Figure 5A shows the dislocation configurations and quantitative Burgers vector statistics for 1B and 5B. In 1B, only a few long, straight dislocations, primarily 1/2­[111] and 1/2­[111], are observed. As Caillard et al. reported, forming ⟨100⟩ dislocations in pure W is energetically unfavorable, resulting in weak interactions and low entanglement between 1/2⟨111⟩ dislocations along different directions.^69^ In contrast, 5B exhibits a significantly higher dislocation density, uniformly distributed along all four 1/2⟨111⟩ directions, enhancing the likelihood of interactions and entanglement. This promotes the formation of numerous low-mobility ⟨100⟩ dislocations. To quantify this contrast, the total dislocation count in 5B is approximately 2.89 times that in 1B (1B: 18 dislocations; 5B: 52 dislocations). Experimentally, at a comparable length scale, the dislocation density calculated for 5B at RT (Figure S9 in the Supporting Information) is 9.8 × 10^16^ m^–2^, about 2.45 times higher than the value reported for 1B at 11% compressive strain (4 × 10^16^ m^–2^) by Wang et al.^70^ This comparable ratio suggests a proportional relationship between the enhanced capability of dislocation nucleation and the degree of lattice distortion. Nevertheless, the slightly higher ratio in MD simulations mainly reflects stronger dislocation starvation in 1B, where dislocations in the defect-free nanoscale cell rapidly annihilate at surfaces due to minimal resistance and the confined size. This artificially reduces dislocation retention in 1B relative to experimental conditions, thereby amplifying the density contrast between 1B and 5B. Figure 5B,C further displays the dislocation configurations, Burgers vectors, and edge/screw character at different strains viewed from ⟨100⟩ stress orientation. At RT, edge dislocations exhibit the highest mobility in 1B, while screw dislocations move at nearly half the velocity (1.25 Å/ps), consistent with trends in BCC metals and previous simulations for pure W under similar stress.^25,44,66,71^ In 5B, however, dislocation motion is markedly hindered by ⟨100⟩ entanglement and lattice distortion. Screw dislocations are further obstructed by persistent cross-kinks, resulting in significantly lower velocities compared to 1B.^72^ This aligns with prior findings that screw dislocations in MoNbTaV and MoNbTaW HEAs consistently exhibit higher glide resistance than edge dislocations across all slip planes.^53^ At HT, edge dislocation velocity in 1B increases modestly, while reduced kink-pair nucleation barriers lead to a more pronounced enhancement in screw dislocation mobility.^45^ Conversely, in 5B, dislocations remain trapped in local energy valleys, and cross-kinks in screw segments are not easily eliminated, even at elevated temperatures. As a result, the increase in dislocation velocities with temperature is significantly smaller in 5B than in 1B.

**Figure 5:**
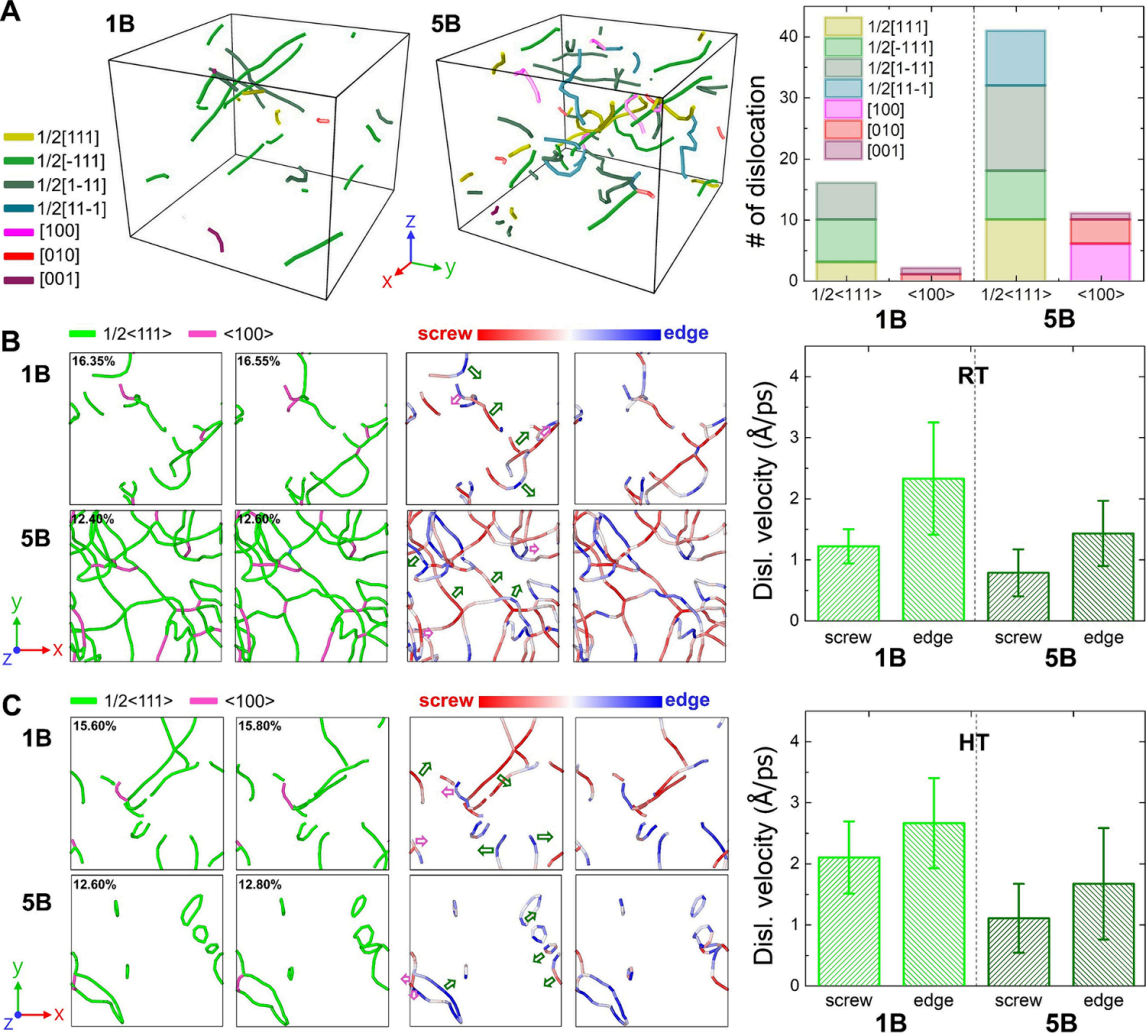
1B and 5B: (A) dislocation configurations with quantitative statistics of Burgers vector; (B–C) dislocation configurations with Burgers vectors and edge/screw character viewed from ⟨100⟩ stress orientation at different strains (green and magenta arrow: moving direction for 1/2⟨111⟩ and ⟨100⟩ dislocation), and the calculated dislocation velocity for 1/2⟨111⟩ dislocations.

In summary, given the pronounced lattice distortion and distinct deformation behaviors across temperatures, WTaMoNbV HEAs were examined in this study. The effect of increasing chemical complexity on mechanical properties and defect evolution was systematically investigated using nanoindentation and microcompression tests under various loading directions and temperatures, supported by TEM observations and MD simulations. For pure tungsten with increasing temperature, strength reduction and stress drops associated with long-range planar slip became progressively evident during deformation. However, complex bonding environments inherent in HEAs substantially diminished orientation-dependent differences and contributed to the retention of high-temperature strength. Moreover, structural complexity also promoted extensive dislocation nucleation across multiple slip systems during elastic-to-plastic transition, while simultaneously restricting edge and screw dislocation motion, thereby resulting in isotropic plastic deformation and significant work hardening. These findings underscore the potential of HEAs as promising candidates for high-temperature structural applications.

## Supplementary Material



























## Notes

Materials and methods; supplementary figures (Figures S1–S12) showing detailed experimental and simulated results (PDF)
Video S1: *In situ* SEM observation on the microcompression of 1B ⟨100⟩ micropillar #1 deformed at RT (MP4)
Video S2: *In situ* SEM observation on the microcompression of 1B ⟨100⟩ micropillar #2 deformed at RT (MP4)
Video S3: *In situ* SEM observation on the microcompression of 1B ⟨100⟩ micropillar #3 deformed at RT (MP4)
Video S4: *In situ* SEM observation on the microcompression of 1B ⟨100⟩ micropillar #4 deformed at RT (MP4)
Video S5: *In situ* SEM observation on the microcompression of 3B ⟨100⟩ micropillar #1 deformed at RT (MP4)
Video S6: *In situ* SEM observation on the microcompression of 3B ⟨100⟩ micropillar #2 deformed at RT (MP4)
Video S7: *In situ* SEM observation on the microcompression of 3B ⟨100⟩ micropillar #3 deformed at RT (MP4)
Video S8: *In situ* SEM observation on the microcompression of 3B ⟨100⟩ micropillar #4 deformed at RT (MP4)
Video S9: *In situ* SEM observation on the microcompression of 5B ⟨100⟩ micropillar #1 deformed at RT (MP4)
Video S10: *In situ* SEM observation on the microcompression of 5B ⟨100⟩ micropillar #2 deformed at RT (MP4)
Video S11: *In situ* SEM observation on the microcompression of 5B ⟨100⟩ micropillar #3 deformed at RT (MP4)
Video S12: *In situ* SEM observation on the microcompression of 5B ⟨100⟩ micropillar #4 deformed at RT (MP4)

## References

[1] Yeh J. W., Chen S. K., Lin S. J., Gan J. Y., Chin T. S., Shun T. T., Tsau C. H., Chang S. Y. (2004). Nanostructured high-entropy
alloys with multiple principal elements: novel alloy design concepts
and outcomes. Adv. Eng. Mater..

[2] Miracle D. B., Senkov O. N. (2017). A critical review
of high entropy alloys and related
concepts. Acta Mater..

[3] George E. P., Raabe D., Ritchie R. O. (2019). High-entropy
alloys. Nature reviews materials.

[4] Yeh J.-W., Lin S.-J., Chin T.-S., Gan J.-Y., Chen S.-K., Shun T.-T., Tsau C.-H., Chou S.-Y. (2004). Formation of simple
crystal structures in Cu-Co-Ni-Cr-Al-Fe-Ti-V alloys with multiprincipal
metallic elements. Metallurgical and Materials
Transactions A.

[5] Wang W.-R., Wang W.-L., Wang S.-C., Tsai Y.-C., Lai C.-H., Yeh J.-W. (2012). Effects of Al addition on the microstructure
and mechanical
property of AlxCoCrFeNi high-entropy alloys. Intermetallics.

[6] Tung C.-C., Yeh J.-W., Shun T.-t., Chen S.-K., Huang Y.-S., Chen H.-C. (2007). On the elemental
effect of AlCoCrCuFeNi high-entropy
alloy system. Materials letters.

[7] Yeh J.-W., Chang S.-Y., Hong Y.-D., Chen S.-K., Lin S.-J. (2007). Anomalous
decrease in X-ray diffraction intensities of Cu-Ni-Al-Co-Cr-Fe-Si
alloy systems with multi-principal elements. Materials chemistry and physics.

[8] Tsai K.-Y., Tsai M.-H., Yeh J.-W. (2013). Sluggish
diffusion in co-cr-fe-mn-ni
high-entropy alloys. Acta Mater..

[9] Zhang Y., Zuo T. T., Tang Z., Gao M. C., Dahmen K. A., Liaw P. K., Lu Z. P. (2014). Microstructures and properties of
high-entropy alloys. Prog. Mater. Sci..

[10] Gao, M. C. ; Yeh, J.-W. ; Liaw, P. K. ; Zhang, Y. High-entropy alloys; Springer International Publishing: Cham, 2016.

[11] Gludovatz B., Hohenwarter A., Catoor D., Chang E. H., George E. P., Ritchie R. O. (2014). A fracture-resistant
high-entropy alloy for cryogenic
applications. Science.

[12] Naeem M., He H., Zhang F., Huang H., Harjo S., Kawasaki T., Wang B., Lan S., Wu Z., Wang F. (2020). Cooperative deformation
in high-entropy alloys at ultralow temperatures. Science advances.

[13] Otto F., Dlouhý A., Somsen C., Bei H., Eggeler G., George E. P. (2013). The influences
of temperature and microstructure on
the tensile properties of a CoCrFeMnNi high-entropy alloy. Acta Mater..

[14] Gludovatz B., Hohenwarter A., Thurston K. V., Bei H., Wu Z., George E. P., Ritchie R. O. (2016). Exceptional damage-tolerance of a
medium-entropy alloy CrCoNi at cryogenic temperatures. Nat. Commun..

[15] Wang S., Wu M., Shu D., Zhu G., Wang D., Sun B. (2020). Mechanical
instability and tensile properties of TiZrHfNbTa high entropy alloy
at cryogenic temperatures. Acta Mater..

[16] Zou Y., Wheeler J. M., Ma H., Okle P., Spolenak R. (2017). Nanocrystalline
high-entropy alloys: a new paradigm in high-temperature strength and
stability. Nano Lett..

[17] Senkov O. N., Wilks G., Scott J., Miracle D. B. (2011). Mechanical properties
of Nb25Mo25Ta25W25 and V20Nb20Mo20Ta20W20 refractory high entropy
alloys. Intermetallics.

[18] Maresca F., Curtin W. A. (2020). Theory of screw
dislocation strengthening in random
BCC alloys from dilute to “High-Entropy” alloys. Acta Mater..

[19] Lee C., Maresca F., Feng R., Chou Y., Ungar T., Widom M., An K., Poplawsky J. D., Chou Y.-C., Liaw P. K. (2021). Strength can be controlled
by edge dislocations in refractory high-entropy alloys. Nat. Commun..

[20] Guo S., Ng C., Lu J., Liu C. (2011). Effect of valence electron
concentration
on stability of fcc or bcc phase in high entropy alloys. Journal of applied physics.

[21] Guo S., Hu Q., Ng C., Liu C. (2013). More than entropy in high-entropy
alloys: Forming solid solutions or amorphous phase. Intermetallics.

[22] Wang Z., Huang Y., Yang Y., Wang J., Liu C. (2015). Atomic-size
effect and solid solubility of multicomponent alloys. Scripta Materialia.

[23] Pickering E., Jones N. (2016). High-entropy alloys:
a critical assessment of their founding principles
and future prospects. International Materials
Reviews.

[24] Zhang Y., Zhou Y. J., Lin J. P., Chen G. L., Liaw P. K. (2008). Solid-solution
phase formation rules for multi-component alloys. Adv. Eng. Mater..

[25] Chen B., Li S., Zong H., Ding X., Sun J., Ma E. (2020). Unusual activated
processes controlling dislocation motion in body-centered-cubic high-entropy
alloys. Proc. Natl. Acad. Sci. U. S. A..

[26] Maresca F., Curtin W. A. (2020). Mechanistic origin of high strength in refractory BCC
high entropy alloys up to 1900K. Acta Mater..

[27] Eleti R. R., Chokshi A. H., Shibata A., Tsuji N. (2020). Unique high-temperature
deformation dominated by grain boundary sliding in heterogeneous necklace
structure formed by dynamic recrystallization in HfNbTaTiZr BCC refractory
high entropy alloy. Acta Mater..

[28] Eleti R. R., Bhattacharjee T., Shibata A., Tsuji N. (2019). Unique deformation
behavior and microstructure evolution in high temperature processing
of HfNbTaTiZr refractory high entropy alloy. Acta Mater..

[29] Guo N., Wang L., Luo L., Li X., Su Y., Guo J., Fu H. (2015). Microstructure
and mechanical properties of refractory
MoNbHfZrTi high-entropy alloy. Materials &
Design.

[30] Liu X., Bai Z., Ding X., Yao J., Wang L., Su Y., Fan Z., Guo J. (2021). A novel light-weight refractory high-entropy alloy
with high specific strength and intrinsic deformability. Mater. Lett..

[31] Owen L. R., Jones N. G. (2018). Lattice distortions in high-entropy alloys. J. Mater. Res..

[32] Wang Z., Pattamatta A. S., Han J., Srolovitz D. J. (2024). Scaling
laws for lattice distortions: Application to high entropy alloys. PNAS nexus.

[33] Li J., Chen Y., He Q., Xu X., Wang H., Jiang C., Liu B., Fang Q., Liu Y., Yang Y. (2022). Heterogeneous lattice strain strengthening in severely
distorted crystalline solids. Proc. Natl. Acad.
Sci. U. S. A..

[34] Beake B. D., Goel S. (2018). Incipient plasticity
in tungsten during nanoindentation: Dependence
on surface roughness, probe radius and crystal orientation. International Journal of Refractory Metals and Hard Materials.

[35] Wang F., Balbus G. H., Xu S., Su Y., Shin J., Rottmann P. F., Knipling K. E., Stinville J.-C., Mills L. H., Senkov O. N. (2020). Multiplicity of dislocation
pathways in a refractory multiprincipal element alloy. Science.

[36] Pisarenko G., Borisenko V., Kashtalyan Y. A. (1964). The effect of temperature on the
hardness and modulus of elasticity of tungsten and molybdenum (20–2700‡). Soviet Powder Metallurgy and Metal Ceramics.

[37] Gibson J. S.-L., Roberts S. G., Armstrong D. E. (2015). High temperature
indentation of helium-implanted
tungsten. Materials Science and Engineering:
A.

[38] Feng R., Feng B., Gao M. C., Zhang C., Neuefeind J. C., Poplawsky J. D., Ren Y., An K., Widom M., Liaw P. K. (2021). Superior High-Temperature
Strength in a Supersaturated
Refractory High-Entropy Alloy. Adv. Mater..

[39] Varvenne C., Luque A., Curtin W. A. (2016). Theory
of strengthening in fcc high
entropy alloys. Acta Mater..

[40] Zou Y., Maiti S., Steurer W., Spolenak R. (2014). Size-dependent plasticity
in an Nb25Mo25Ta25W25 refractory high-entropy alloy. Acta Mater..

[73] Donachie, M. J. ; Donachie, S. J. Superalloys: A technical guide; ASM international, 2002.

[74] Bhujangrao T., Veiga F., Suarez A., Iriondo E., Mata F. G. (2020). High-temperature
mechanical properties of IN718 alloy: Comparison of additive manufactured
and wrought samples. Crystals.

[75] Harris K., Erickson G. L., Sikkenga S. L., Brentnall W. D., Aurrecoechea J. M., Kubarych K. G. (1993). Development of two
rhenium-containing
superalloys for single-crystal blade and directionally solidified
vane applications in advanced turbine engines.. J. Mat. Eng. Perf..

[76] Juan C.-C., Tsai M.-H., Tsai C.-W., Lin C.-M., Wang W.-R., Yang C.-C., Chen S.-K., Lin S.-J., Yeh J.-W. (2015). Enhanced
mechanical properties of HfMoTaTiZr and HfMoNbTaTiZr refractory high-entropy
alloys. Intermetallics.

[77] Senkov O. N., Scott J. M., Senkova S. V., Meisenkothen F., Miracle D. B., Woodward C. F. (2012). Microstructure and
elevated temperature
properties of a refractory TaNbHfZrTi alloy. J. Mat. Sci..

[78] Jha, S. ; Muskeri, S. ; Yang, Y. C. ; Sadeghilaridjani, M. ; Bhowmick, S. ; Mukherjee, S. Small-Scale Deformation Behavior of Refractory High Entropy Alloy as a Function of Strain Rate and Temperature. Available at SSRN: https://ssrn.com/abstract=4001342 or 10.2139/ssrn.4001342.

[79] Senkov O. N., Woodward C., Miracle D. B. (2014). Microstructure
and properties of
aluminum-containing refractory high-entropy alloys. JOM.

[80] Senkov O.N., Senkova S.V., Miracle D.B., Woodward C. (2013). Mechanical
properties
of low-density, refractory multi-principal element alloys of the Cr-Nb-Ti-V-;Zr
system. Materials Science and Engineering: A.

[41] Hu Y., Shu L., Yang Q., Guo W., Liaw P. K., Dahmen K. A., Zuo J.-M. (2018). Dislocation avalanche
mechanism in slowly compressed
high entropy alloy nanopillars. Communications
Physics.

[42] Rizzardi Q., Derlet P., Maaß R. (2021). Microstructural signatures of dislocation
avalanches in a high-entropy alloy. Physical
review materials.

[43] Wei S., Zhao Y., Jang J.-i., Ramamurty U. (2022). Rate-dependent
mechanical behavior of single-, bi-, twinned-, and poly-crystals of
CoCrFeNi high-entropy alloy. Journal of Materials
Science & Technology.

[44] Chaussidon J., Fivel M., Rodney D. (2006). The glide of screw
dislocations in
bcc Fe: atomistic static and dynamic simulations. Acta Mater..

[45] Schneider A., Kaufmann D., Clark B., Frick C., Gruber P., Mönig R., Kraft O., Arzt E. (2009). Correlation
between
critical temperature and strength of small-scale bcc pillars. Physical review letters.

[46] Wang W. Y., Shang S. L., Wang Y., Han F., Darling K. A., Wu Y., Xie X., Senkov O. N., Li J., Hui X. D. (2017). Atomic and electronic basis for the serrations
of refractory high-entropy
alloys. npj Computational Materials.

[47] Liu X., Hua D., Wang W., Zhou Q., Li S., Shi J., He Y., Wang H. (2022). Atomistic understanding of incipient plasticity in
BCC refractory high entropy alloys. J. Alloys
Compd..

[48] Utt D., Lee S., Xing Y., Jeong H., Stukowski A., Oh S. H., Dehm G., Albe K. (2022). The origin of jerky
dislocation motion in high-entropy alloys. Nat.
Commun..

[49] Brechtl J., Chen S., Lee C., Shi Y., Feng R., Xie X., Hamblin D., Coleman A. M., Straka B., Shortt H. (2020). A review of the serrated-flow phenomenon and its role in the deformation
behavior of high-entropy alloys. Metals.

[50] Torrents
Abad O., Wheeler J. M., Michler J., Schneider A. S., Arzt E. (2016). Temperature-dependent size effects on the strength of Ta and W micropillars. Acta Mater..

[51] Ghafarollahi A., Curtin W. A. (2022). Screw-controlled strength of BCC
non-dilute and high-entropy
alloys. Acta Mater..

[52] Wang X., Maresca F., Cao P. (2022). The hierarchical energy
landscape
of screw dislocation motion in refractory high-entropy alloys. Acta Mater..

[53] Romero R. A., Xu S., Jian W.-R., Beyerlein I. J., Ramana C. (2022). Atomistic simulations
of the local slip resistances in four refractory multi-principal element
alloys. International Journal of Plasticity.

[54] Yin S., Ding J., Asta M., Ritchie R. O. (2020). Ab initio modeling
of the energy landscape for screw dislocations in body-centered cubic
high-entropy alloys. npj Computational Materials.

[55] Chen B., Li S., Ding J., Ding X., Sun J., Ma E. (2023). Correlating
dislocation mobility with local lattice distortion in refractory multi-principal
element alloys. Scripta Materialia.

[56] Lee C., Maresca F., Feng R., Chou Y., Ungar T., Widom M., An K., Poplawsky J. D., Chou Y.-C., Liaw P. K. (2021). Strength
can be controlled
by edge dislocations in refractory high-entropy alloys. Nat. Commun..

[57] Vitek V. (1992). Structure
of dislocation cores in metallic materials and its impact on their
plastic behaviour. Prog. Mater. Sci..

[58] Argon, A. Strengthening mechanisms in crystal plasticity; OUP Oxford, 2007.

[59] Srivastava, K. Atomistically-informed discrete dislocation dynamics modeling of plastic flow in body-centered cubic metals. Dissertation, Karlsruher Institut für Technologie, 2014. 10.5445/IR/1000042367

[60] Bulatov V. V., Hsiung L. L., Tang M., Arsenlis A., Bartelt M. C., Cai W., Florando J. N., Hiratani M., Rhee M., Hommes G. (2006). Dislocation multi-junctions and strain hardening. Nature.

[61] Alhafez I. A., Deluigi O. R., Tramontina D., Merkert N., Urbassek H. M., Bringa E. M. (2024). Nanoindentation
into a bcc high-entropy HfNbTaTiZr
alloy—an atomistic study of the effect of short-range order. Sci. Rep..

[62] Yin Y.-Z., An Y., Ding J., Han W.-Z. (2025). Dislocation
Multijunction-Driven
Plasticity in HfNbTiZr High-Entropy Alloys. Nano Lett..

[63] Shao Y.-T., Yuan R., Hu Y., Yang Q., Zuo J.-M. (2019). The paracrystalline
nature of lattice distortion in a high entropy alloy. arXiv.

[64] Zhu T., Li J. (2010). Ultra-strength materials. Prog. Mater. Sci..

[65] Li J., Van Vliet K. J., Zhu T., Yip S., Suresh S. (2002). Atomistic
mechanisms governing elastic limit and incipient plasticity in crystals. Nature.

[66] Stukowski A., Cereceda D., Swinburne T. D., Marian J. (2015). Thermally-activated
non-Schmid glide of screw dislocations in W using atomistically-informed
kinetic Monte Carlo simulations. International
Journal of Plasticity.

[67] Yin S., Zuo Y., Abu-Odeh A., Zheng H., Li X.-G., Ding J., Ong S. P., Asta M., Ritchie R. O. (2021). Atomistic
simulations
of dislocation mobility in refractory high-entropy alloys and the
effect of chemical short-range order. Nat. Commun..

[68] Zhang L., Xiang Y., Han J., Srolovitz D. J. (2019). The effect
of randomness on the strength of high-entropy alloys. Acta Mater..

[69] Caillard D., Bienvenu B., Clouet E. (2022). Anomalous slip in body-centred
cubic
metals. Nature.

[70] Wang J., Wang Y., Cai W., Li J., Zhang Z., Mao S. X. (2018). Discrete shear band plasticity through
dislocation
activities in body-centered cubic tungsten nanowires. Sci. Rep..

[71] Marian J., Cai W., Bulatov V. V. (2004). Dynamic transitions
from smooth to rough to twinning
in dislocation motion. Nature materials.

[72] Zhou X., He S., Marian J. (2021). Cross-kinks
control screw dislocation strength in equiatomic
bcc refractory alloys. Acta Mater..

