# Supplementary material for: Small-Scale Insight into Uniform Deformability and Softening Resistance of Refractory High-Entropy Alloy
Source: Nano Lett. 2026 Feb 19;26(8):2825–34. doi: 10.1021/acs.nanolett.5c05077 (PMC12964539; doi:10.1021/acs.nanolett.5c05077)
Supplement: Supplementary file 1 [file nl5c05077_si_001.pdf]

SUPPORTING INFORMATION

Small-Scale Insight into Uniform Deformability and Softening Resistance of Refractory High-Entropy Alloy

*Cheng-Yuan Tsai[1], Wen-Ju Chen[1], Yuan-Tao Hsu[1], Chi-Huan Tung[1], Su-Jien Lin[1,2], Jien-Wei Yeh[1,2], and Shou-Yi Chang[1,2,]**

[1] Department of Materials Science and Engineering, National Tsing Hua University, Hsinchu 30013, Taiwan

[2] High Entropy Materials Center, National Tsing Hua University, Hsinchu, Taiwan

*Corresponding author's email address: changsy@mx.nthu.edu.tw

Materials and Methods

Sample Preparation and Structural Analysis. In this study, W (1B) was selected as the base element, and Ta, Mo, Nb, and V were sequentially added to design the WTa (2B), WTaMo (3B), WTaMoNb (4B), and WTaMoNbV (5B) alloys. All the alloys were prepared via vacuum arc melting and remelted at least five times to ensure compositional homogeneity. The ingots were subsequently encapsulated in double-layer quartz tubes and subjected to the homogenization at 1400°C for 24 h, followed by furnace cooling. Electron probe microanalysis (EPMA, JEOL JXA−8500F) was used to verify chemical compositions of the alloys. Moreover, the theoretical lattice parameter, density, lattice distortion, and Young's modulus of the 1B–5B alloys were calculated based on the rule of mixtures by incorporating data from the Metal Handbook database (Tables S1 and S2 in the Supporting Information). X-ray diffraction (XRD, Bruker D8) was used to analyze crystal structures and lattice parameters. Electron backscattered diffraction (EBSD, Carl Zeiss AG-SUPRA 55VP) was applied to acquire grain orientations (step size: 5 μm; current and voltage: 15 nA and 20 kV).

Nanoindentation. A nanoindenter (Hysitron TI 980) with an xSol® heating/cooling stage and a Berkovich diamond indenter tip (TI-0283, area function calibrated) was implemented to measure the Young's modulus and hardness of the 1B, 3B, and 5B in ⟨100⟩ and ⟨111⟩ grains at −80°C (LT), 25°C (RT), and 300°C (HT). An advanced XPM (accelerated property mapping) mode was applied to avoid sample oxidation and thermal drift (maximum load of 8000 μN, loading–holding–unloading in 0.2–0.1–0.1 s; at least 60 measurements being conducted for each crystal orientation). The degree of anisotropy was quantified using the elastic ($A_E = E_{111}/E_{100}$, where E_{100} and E_{111} denote Young's modulus in ⟨100⟩ and ⟨111⟩ grains, respectively) and plastic ($A_H = H_{111}/H_{100}$, where H_{100} and H_{111} denote hardness in ⟨100⟩ and ⟨111⟩ grains, respectively) anisotropy indices, together with the anisotropy reduction ratio defined as: $R = (|A_{baseline} - 1| - |A_{alloy} - 1|)/|A_{baseline} - 1| \times 100\%$, where 1B was taken as the baseline and $|A - 1|$ quantifies the deviation from isotropy.

Microcompression. Focused ion beam (FIB, FEI Helios 600i, Hitachi NX2000, and FEI Helios G3CX) was utilized to fabricate micropillars (diameter: 2 μm; height: 5−6 μm) in ⟨100⟩ grains of the 1B, 3B, and 5B alloys. In addition, FIB was also employed to prepare TEM lamella (length and width: ~6 μm; thickness: ~50 nm) from the longitudinally sectioned deformed micropillars. For microcompression for the 1B, 3B, and 5B micropillars along ⟨100⟩ stress orientation at different temperatures, a picoindenter (Hysitron PI85) integrated with a scanning electron microscope (SEM, NOVA NANO SEM 450) was implemented to perform *in-situ* microcompression at 25°C (RT) (total displacement: 1 μm; compression rate: 4 nm/s); a nanoindenter (Hysitron TI 980) with an xSol® heating/cooling stage and a flat-ended sapphire indenter tip was used at −80°C (LT) and 300°C (HT) (total strain: 20%; compression rate: 4 nm/s). The deformed micropillars (1B and 3B at RT; 5B across all the temperatures) were further analyzed using high-resolution and scanning transmission electron microscopy (TEM, JEOL JEM−F200) to examine dislocation and lattice structures. For identifying dislocation character, the observed line contrast in TEM images was compared with the projected theoretical dislocation lines on the viewing plane for $\vec{b} = 1/2\langle 111\rangle$. Edge segments exhibit line directions perpendicular to $\vec{b}$ (along ⟨112⟩), whereas screw segments lie parallel to $\vec{b}$ (along ⟨111⟩). Regions with blurred contrast or line directions deviating by more than 1° were excluded and classified as unknown defect regions or preparation-induced artifacts to ensure accurate counting of dislocations of each character. Strain analysis was also performed by using the Strain++ software equipped with geometric phase analysis (GPA) to visualize strain distribution.[1]

Molecular Dynamics (MD) Simulations. The MD simulations on 1B and 5B of compressive deformation were performed by using the LAMMPS package.[2] For 1B, the second nearest neighbor modified embedded-atom method (2NN MEAM) interatomic potential was adopted to describe atomic interactions.[3] For 5B, a machine learning-based tabulated Gaussian approximation potential (tabGAP) was utilized.[4] The simulation cells for 1B and 5B were constructed as defect-free BCC crystals with dimensions of 9×9×9 nm^3 along the x [100], y [010], and z [001] directions, containing 54,000 atoms in total. Periodic boundary conditions were applied to the three axes. Each simulation cell was energetically minimized by using the conjugate gradient method (CG) to obtain a stress-free configuration. An NPT ensemble was applied at LT, RT, and HT for 40

ps (timestep: 2 fs) to achieve thermal equilibrium. Uniaxial compressive loading along the z-axis was performed under the NVT ensemble at a constant engineering strain rate of $5\times10^8 s^{-1}$ to a total strain 25%. The von Mises stress was calculated using the Virial stress formulation to generate stress-strain curves. Dislocation structures were further analyzed using the dislocation extraction algorithm (DXA)[5] implemented in the OVITO visualization software.

Table S1. Fundamental properties of constituent elements in 1B−5B alloys.

	W	Ta	Mo	Nb	V
Radius R (Å)	1.37	1.43	1.36	1.42	1.31
Modulus E (GPa)	411	186	329	103	128
Density ρ (g/cm^3)	19.25	16.60	10.22	8.57	6.16
Melting point (^{O}C)	3422	3017	2623	2477	1910

ΔH (KJ/Mole)					
	W	Ta	Mo	Nb	V
W	0	−11	0	−13	−1
Ta	−11	0	−7	0	−1
Mo	0	−7	0	−9	0
Nb	−13	0	−9	0	−2
V	−1	−2	0	−1	0

Fusion Heat (KJ/Mole)					
	W	Ta	Mo	Nb	V
W	35.30	–	–	–	–
Ta	41.44	36.57	–	–	–
Mo	36.39	40.53	37.48	–	–
Nb	39.15	33.29	38.24	30.00	–
V	28.90	30.04	29.49	26.25	21.50

Table S2. Chemical compositions and theoretical properties of 1B−5B alloys calculated using the rule of mixtures.

(at%)	1B	2B	3B	4B	5B
W	98.899	49.637	32.855	26.047	20.238
Ta	–	48.759	33.395	25.641	21.582
Mo	–	–	32.436	22.011	18.863
Nb	–	–	–	23.269	19.581
V	–	–	–	–	19.709
O	1.101	1.604	1.314	3.032	0.027
Radius R (Å)	1.37	1.40	1.39	1.40	1.38
Lattice Constant a (Å)	3.165	3.232	3.203	3.223	3.185
Density ρ (g/cm^3)	19.04	17.67	15.20	13.56	12.30
Atomic size difference δ (%)[#1]	0	2.581	2.565	3.619	3.617
Parameter of lattice distortion δ_l (%)[#2]	0.90	1.30	1.29	1.31	1.31
Modulus E (GPa)	411	300	308	259	231
ΔH (KJ/Mole)	0	−5.499	−3.996	−4.569	−3.555
Fusion Heat (KJ/Mole)	35.30	38.68	38.46	37.39	34.00

#1 Atomic size difference $\delta = \sqrt{\sum_{i=1}^{n} C_i(1 - r_i/\bar{r})^2}$ (n: number of the elements; C_i: atomic ratio of the i-th element; r_i: radius of the i-th element; averaged radius $\bar{r} = \sum_{i=1}^{n} C_i r_i$).

#2 Parameter of lattice distortion δ_l was determined and averaged from three selected (hkl) reflections at different Bragg angles. For each peak, δ_l was calculated using $\delta_l = \Delta d_{(hkl)}/d_{(hkl)} \approx \beta/4\tan(\theta)$ ($d_{(hkl)}$ and $\Delta d_{(hkl)}$: d-spacing of the (hkl) plane and its variation; β: FWHM of the corresponding XRD peak; θ: Bragg angle). This relation follows from the Williamson-Hall approach and provides an upper-limit estimate of lattice distortion.

Table S3. Lattice constants determined from the XRD analysis ($a_{Avg.}$) and rule of mixtures (a_{Theory}), as well as the corresponding deviation (Δa) of 1B–5B alloys.

	Plane	2θ (°)	a (Å)	$a_{Avg.}$ (Å)[#1]	a_{Theory} (Å)	Δa (%)[#2]
	(110)	40.279	3.162			
1B	(200)	58.258	3.164	3.162	3.165	–0.095
	(211)	73.298	3.159			
	(110)	39.375	3.232			
2B	(200)	56.972	3.229	3.229	3.232	–0.093
	(211)	71.515	3.228			
	(110)	39.745	3.203			
3B	(200)	57.629	3.195	3.198	3.203	–0.156
	(211)	72.309	3.197			
	(110)	39.443	3.227			
4B	(200)	57.094	3.223	3.224	3.223	0.031
	(211)	71.648	3.222			
	(110)	39.840	3.196			
5B	(200)	57.703	3.191	3.192	3.185	0.220
	(211)	72.521	3.189			

#1 $a_{Avg.} = (a_{110} + a_{200} + a_{211})/3$

#2 $\Delta a\ (\%) = (a_{Avg.} - a_{Theory})/a_{Theory} \times 100\%$

Table S4. Averaged Young's modulus and hardness of the 1B, 3B, and 5B alloys along ⟨100⟩ and ⟨111⟩ stress orientations acquired by instrumented nanoindentations at different temperatures, with the corresponding elastic (A_E) and plastic anisotropy (A_H) and anisotropy reduction ratio (R).

Young's modulus E (GPa)					
		⟨100⟩	⟨111⟩	A_E	R
1B	LT	400.285	363.602	0.908	–
	RT	317.008	277.496	0.875	–
	HT	229.701	216.844	0.944	–
3B	LT	276.459	266.864	0.965	62.1%
	RT	251.575	227.444	0.904	23.0%
	HT	222.623	206.795	0.929	–27.0%
5B	LT	226.821	220.631	0.973	70.2%
	RT	201.683	201.017	0.997	97.4%
	HT	176.276	181.550	1.030	153.5%
Hardness H (GPa)					
		⟨100⟩	⟨111⟩	A_H	R
1B	LT	8.584	8.934	1.041	–
	RT	6.656	7.267	1.092	–
	HT	4.345	4.801	1.105	–
3B	LT	7.803	7.343	0.941	–44.4%
	RT	6.820	6.231	0.914	6.0%
	HT	6.390	5.824	0.911	15.7%
5B	LT	7.561	7.473	0.988	71.2%
	RT	6.963	7.043	1.012	86.6%
	HT	5.985	5.956	0.995	105.5%

Table S5. Comparison of yield strength and strength retention ratio of the 5B alloy with other state-of-the-art refractory high-entropy alloys and conventional superalloys at various temperatures.

Alloy system	Microstructure	Test temperature (°C)	Yield strength (MPa)	Strength retention ratio[#1]
5B (this work)	SX[#2] ⟨100⟩	RT	1744	1.000
		300	1193	0.684
MoNbTaVW[8]	Polycrystalline (HIP + A[#2])	RT	1246	1.000
		1000	842	0.676
		1400	735	0.590
		1600	656	0.526
MoNbTaW[8]	Polycrystalline (HIP + A)	RT	1058	1.000
		1000	548	0.518
		1400	500	0.473
		1600	405	0.383
HfTaTiVZr[9]	SX ⟨110⟩	RT	~1250	1.000
		200	~900	0.720
		400	~1400	1.120
HfTaTiVZr[9]	SX ⟨111⟩	RT	~1600	1.000
		200	~1000	0.625
		400	~1250	0.781
		800	~900	0.563

Table S5. (Continued) Comparison of yield strength and strength retention ratio of the 5B alloy with other state-of-the-art refractory high-entropy alloys and conventional superalloys at various temperatures.

HfNbTaTiZr[10]	Polycrystalline (HIP + A)	RT	929	1.000
		400	790	0.850
		600	675	0.727
		800	535	0.576
		1000	295	0.318
		1200	92	0.099
HfMoTaTiZr[11]	Polycrystalline (As cast)	RT	1600	1.000
		800	1045	0.653
		1000	855	0.534
		1200	404	0.253
HfMoNbTaTiZr[11]	Polycrystalline (As cast)	RT	1512	1.000
		800	1007	0.666
		1000	814	0.538
		1200	556	0.368
NbTiVZr[12]	Polycrystalline (HIP + A)	RT	1105	1.000
		600	834	0.755
		800	187	0.169
		1000	58	0.052
CrNbTiZr[12]	Polycrystalline (HIP + A)	RT	1260	1.000
		600	1035	0.821
		800	300	0.238
		1000	115	0.091

Table S5. (Continued) Comparison of yield strength and strength retention ratio of the 5B alloy with other state-of-the-art refractory high-entropy alloys and conventional superalloys at various temperatures.

$AlNb_{1.5}Ta_{0.5}Ti_{1.5}Zr_{0.5}$[13]	Polycrystalline (HIP + A)	RT	1280	1.000
		800	728	0.569
		1000	403	0.315
$Al_{0.4}Hf_{0.6}NbTaTiZr$[13]	Polycrystalline (HIP + A)	RT	1841	1.000
		800	796	0.432
		1000	298	0.162
CMSX-4[14]	SX ⟨100⟩	RT	~1100	1.000
		750	~1120	1.018
		950	~950	0.864
		1100	~550	0.500
Mar-M247[15]	Polycrystalline (As cast)	RT	~860	1.000
		760	~850	0.988
		870	~780	0.907
		980	~450	0.523
Inconel 718[16]	Polycrystalline (Wrought)	RT	580	1.000
		650	510	0.879
		1200	150	0.259

#1 Calculated as the ratio of the yield strength at the specific test temperature to the yield strength at room temperature (RT).

#2 SX: Single crystal; HIP: Hot isostatically pressed; A: Annealed

Note: Data for the 5B alloy and refractory high-entropy alloys are obtained from compression tests, while data for superalloys corresponds to tensile yield strengths.

Table S6. Slip system, Schmid factor and angle for BCC-structured ⟨100⟩ micropillars (only consider ⟨111⟩ slip vectors).

Slip system	Schmid factor	Angle[#]
$(110)[\bar{1}11]$	0.408	45°
$(112)[11\bar{1}]$	0.236	35°
$(211)[\bar{1}11]$	0.471	55°
$(123)[11\bar{1}]$	0.154	16°
$(213)[11\bar{1}]$	0.308	32°
$(312)[\bar{1}11]$	0.463	53°

Angle between slip plane and ⟨100⟩ stress orientation.

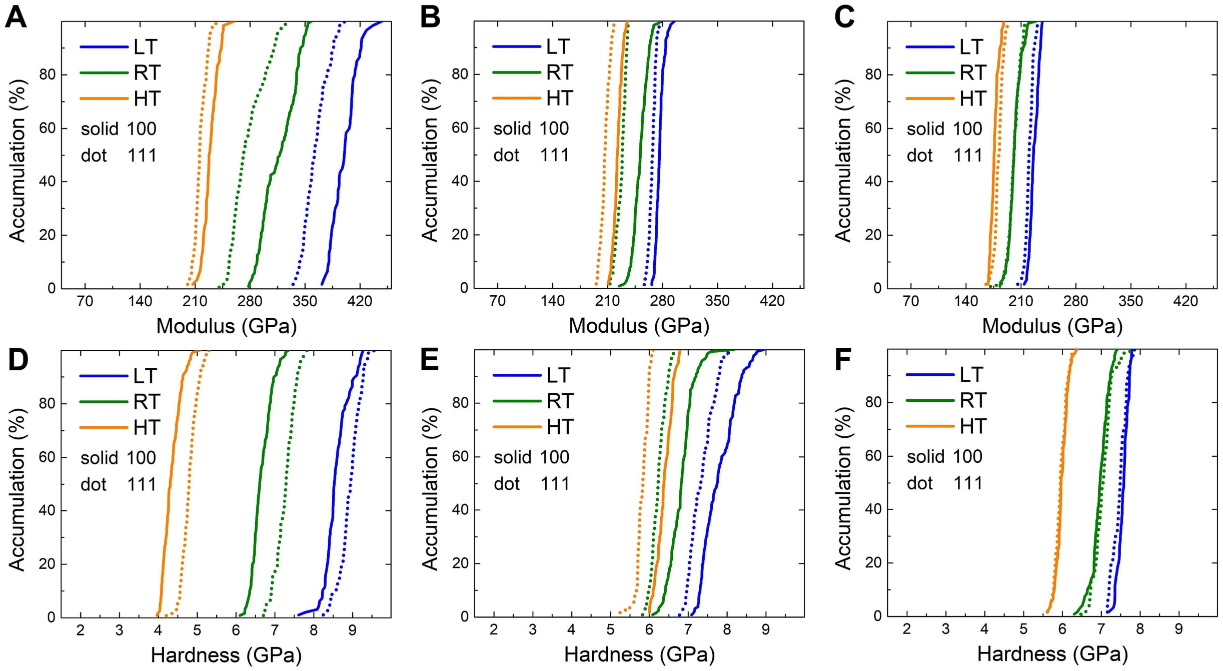

Figure S1. Cumulative plots of Young's modulus and hardness for differently-oriented grains of different samples acquired by instrumented nanoindentations at different temperatures: (A)(D) 1B, (B)(E) 3B, and (C)(F) 5B (solid and dotted line: ⟨100⟩ and ⟨111⟩ stress orientations).

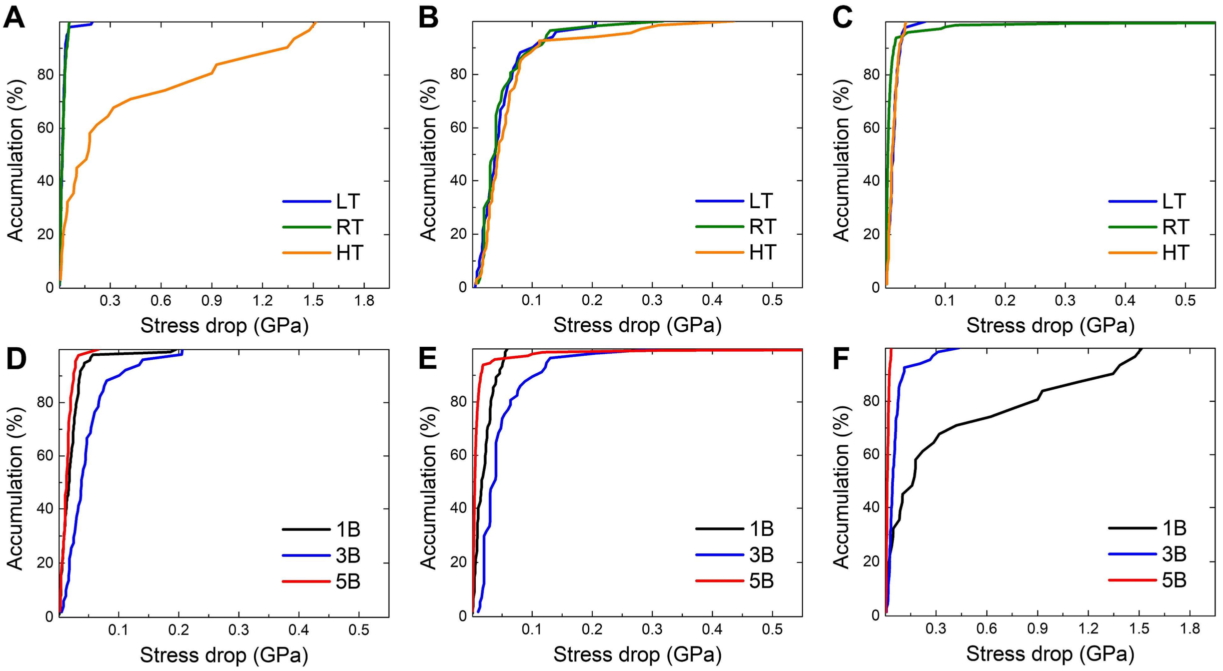

Figure S2. Cumulative plots of stress drop for ⟨100⟩ micropillars of different samples at different temperatures: (A) 1B, (B) 3B, and (C) 5B; (D) LT, (E) RT, and (F) HT.

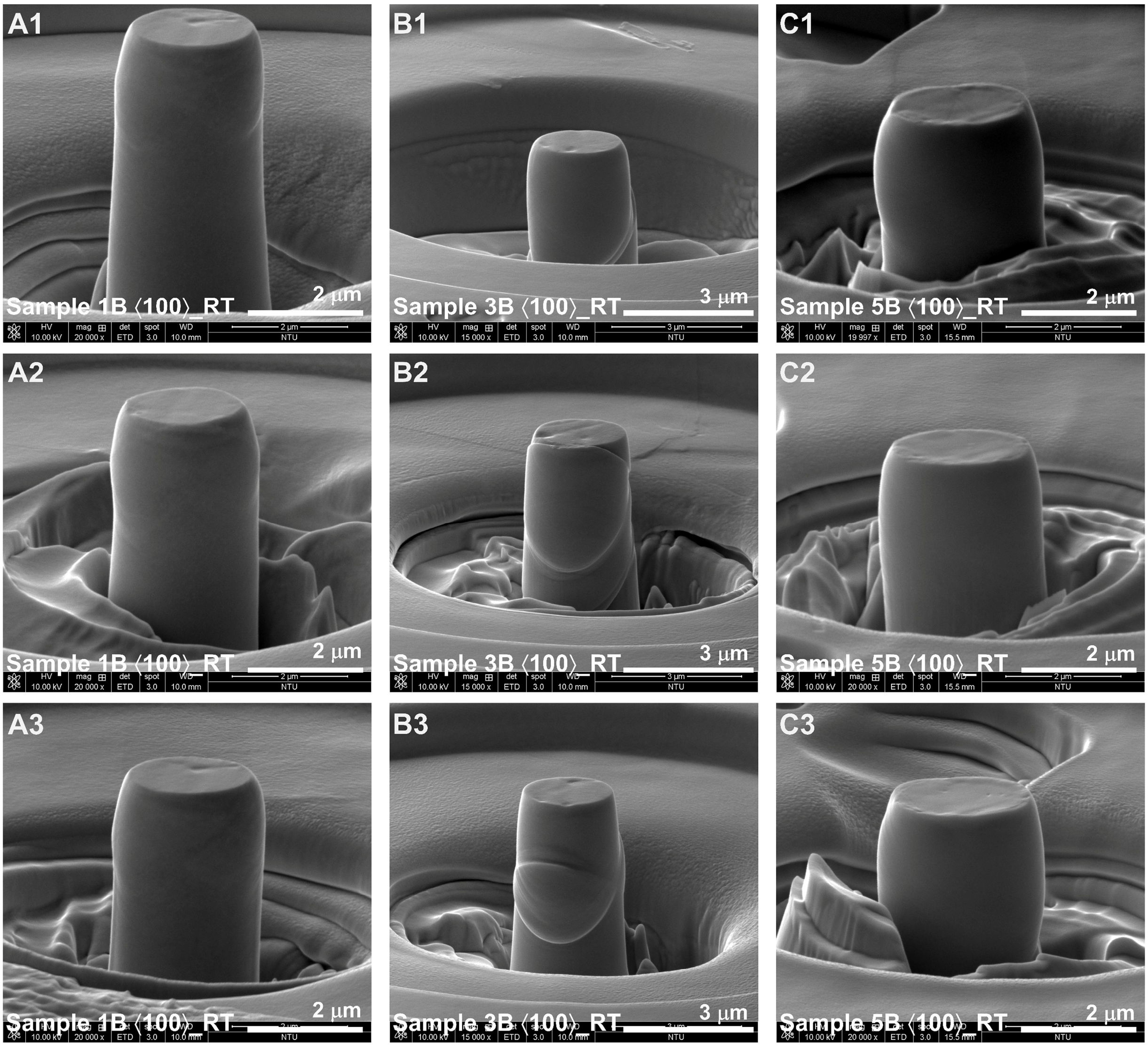

Figure S3. Post-compression SEM micrographs for ⟨100⟩ micropillars (#2 to #4 from top to bottom) deformed at RT: (A) 1B, (B) 3B, and (C) 5B.

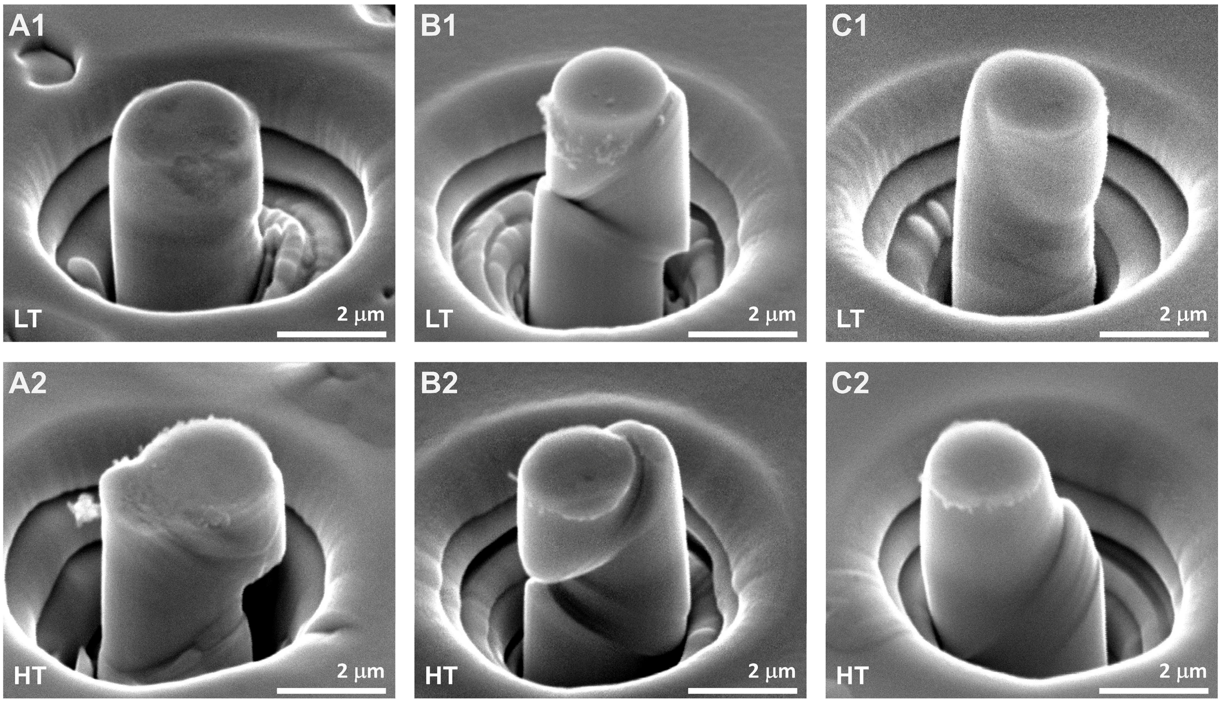

Figure S4. Post-compression SEM micrographs for ⟨100⟩ micropillars deformed at LT and HT: (A) 1B, (B) 3B, and (C) 5B.

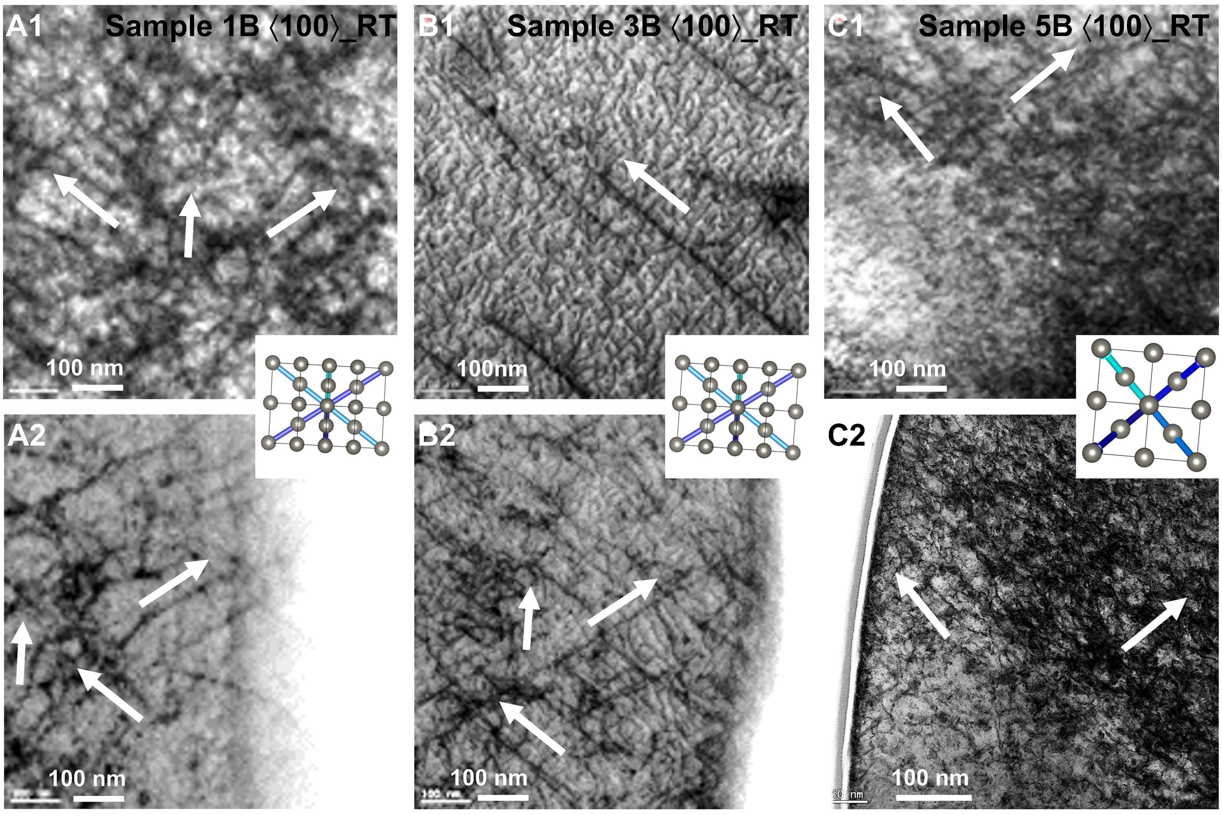

Figure S5. Magnified STEM images of deformation zones 1 and 2 in the longitudinal cross-section for ⟨100⟩ micropillars deformed at room temperature: (A) 1B, (B) 3B, and (C) 5B (corresponding stereographic projections along zone axes [011] and [001] are shown; white arrow: dislocation Burgers vector).

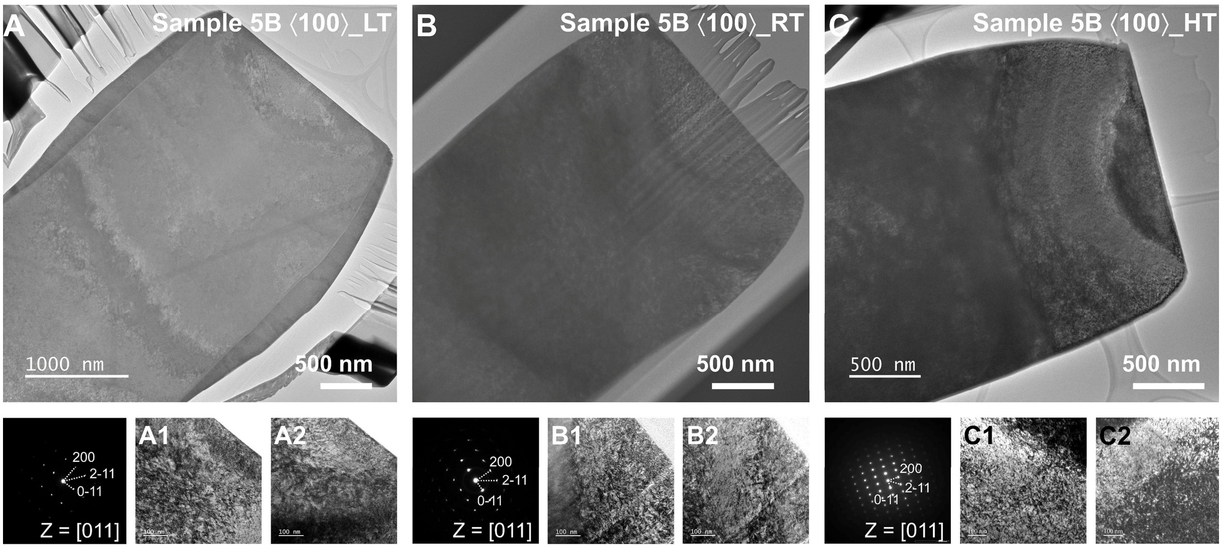

Figure S6. Cross-sectional STEM images, including selected-area diffraction patterns and magnified views of deformation zones 1 and 2, for 5B ⟨100⟩ micropillars deformed at different temperatures: (A) LT, (B) RT, and (C) HT.

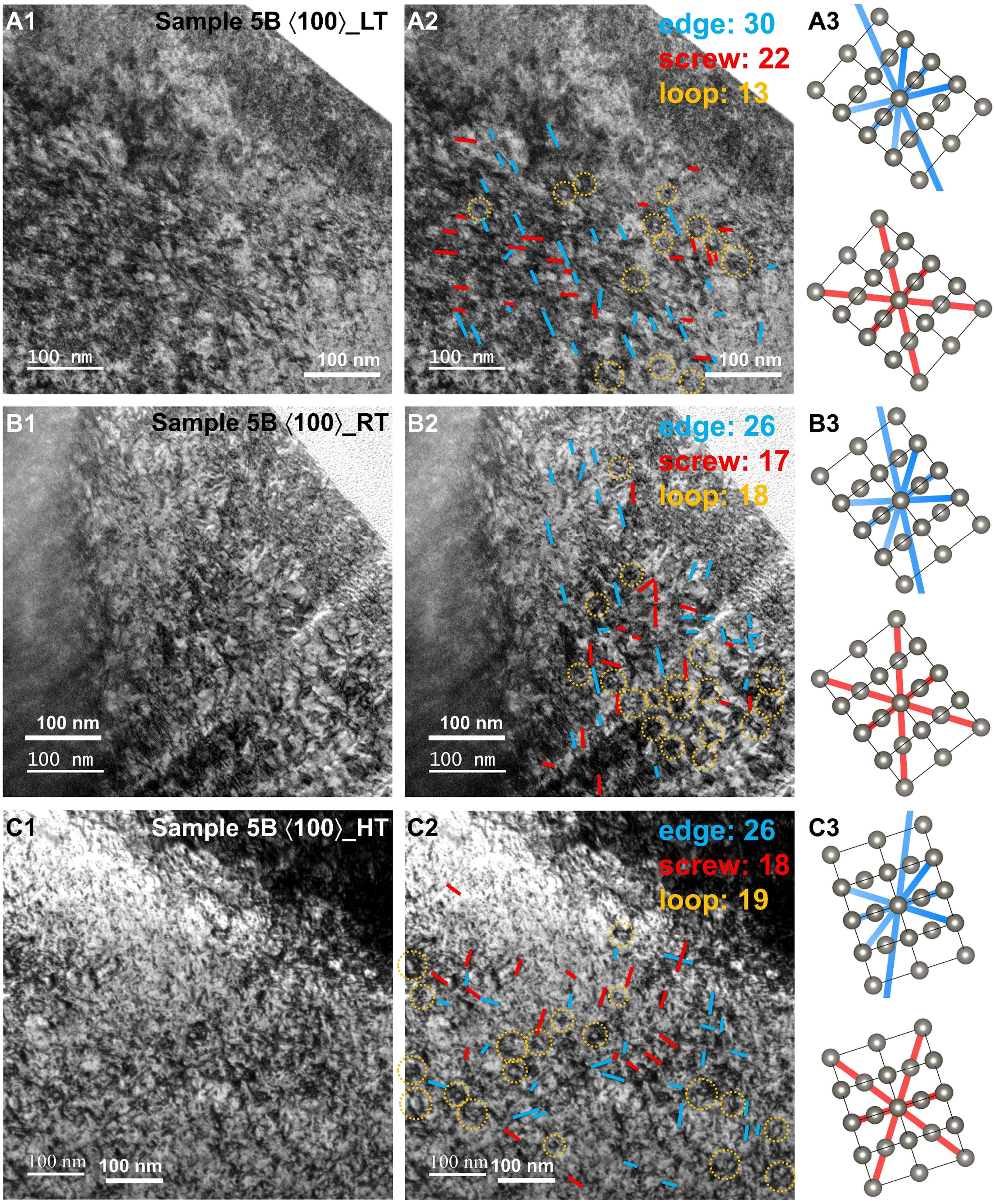

Figure S7. Magnified cross-sectional views of the deformation zone 1 for 5B ⟨100⟩ micropillars deformed at different temperatures with stereographic projections along [011] zone axis: (A) LT, (B) RT, and (C) HT (blue and red lines: 1/2⟨111⟩ edge and screw dislocation lines along ⟨112⟩ and ⟨111⟩ directions individually; yellow dotted circle: dislocation loop; number: dislocation counts).

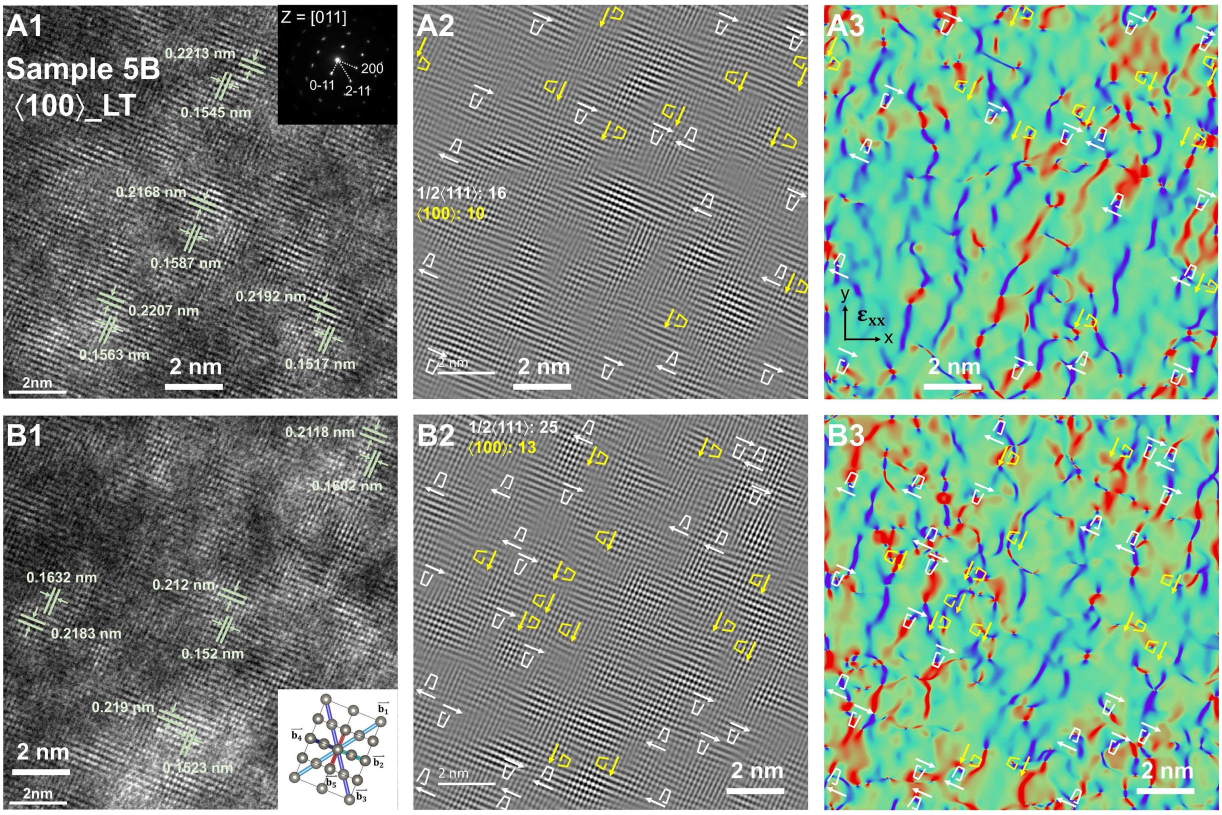

Figure S8. Atomic-resolution filtered iFFT lattice images, and corresponding strain fields (blue: compressive; red: tensile) for 5B ⟨100⟩ micropillars deformed at LT: (A) deformation zone 1, and (B) deformation zone 2 (selected-area diffraction patterns and stereographic projections along zone axes are included) ($\overrightarrow{b_1}$ to $\overrightarrow{b_4}$: ⟨111⟩ directions; $\overrightarrow{b_5}$: ⟨100⟩ directions) (arrow: dislocation slip direction; numbers: dislocation counts for 1/2⟨111⟩ and ⟨001⟩ Burgers vector).

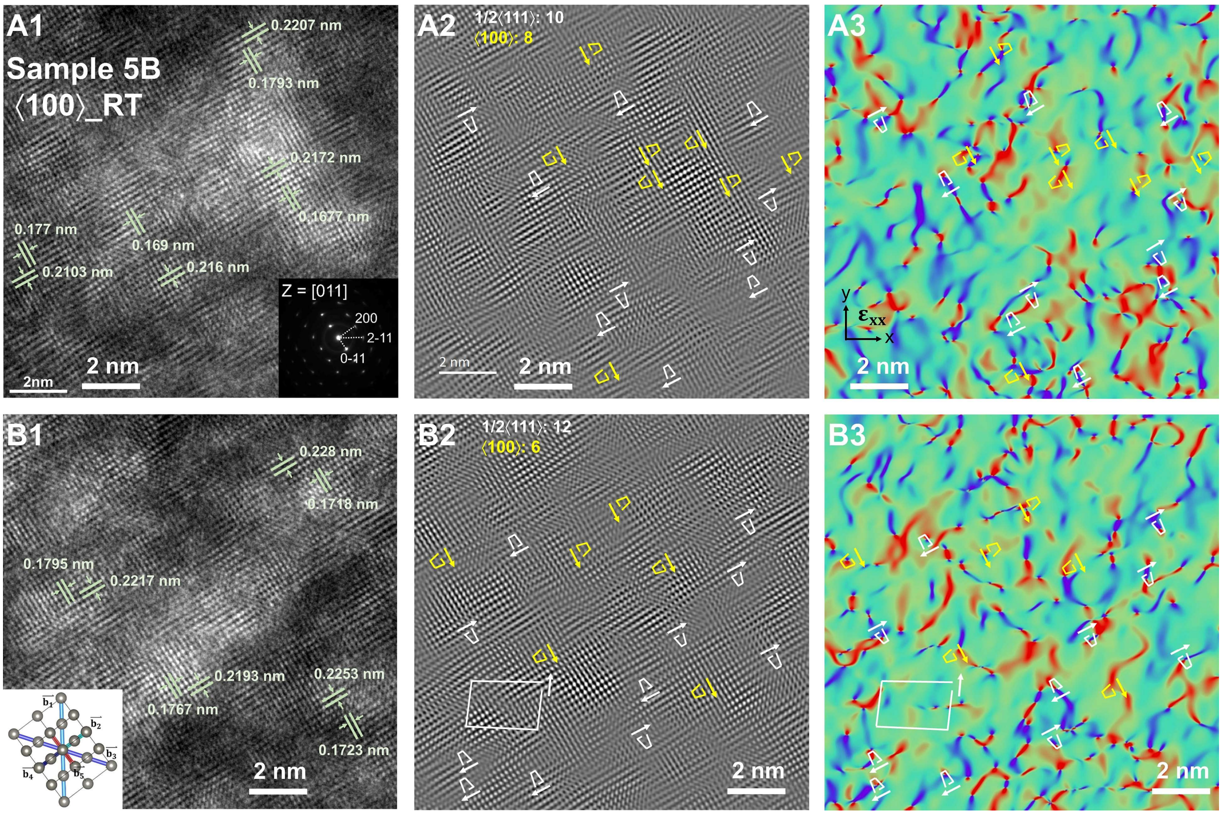

Figure S9. Atomic-resolution filtered iFFT lattice images, and corresponding strain fields (blue: compressive; red: tensile) for 5B ⟨100⟩ micropillars deformed at RT: (A) deformation zone 1, and (B) deformation zone 2 (selected-area diffraction patterns and stereographic projections along zone axes are included) ($\overrightarrow{b_1}$ to $\overrightarrow{b_4}$: ⟨111⟩ directions; $\overrightarrow{b_5}$: ⟨100⟩ directions) (arrow: dislocation slip direction; numbers: dislocation counts for 1/2⟨111⟩ and ⟨001⟩ Burgers vector).

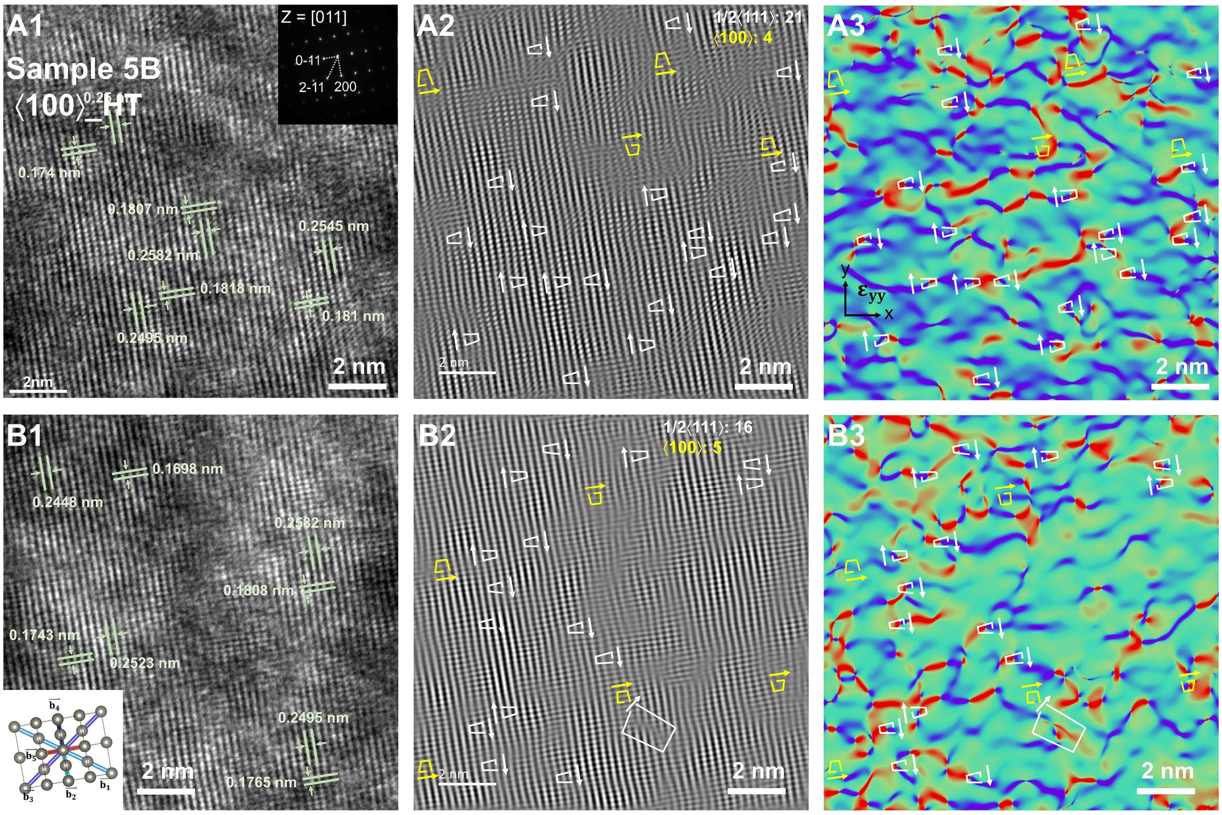

Figure S10. Atomic-resolution filtered iFFT lattice images, and corresponding strain fields (blue: compressive; red: tensile) for 5B ⟨100⟩ micropillars deformed at HT: (A) deformation zone 1, and (B) deformation zone 2 (selected-area diffraction patterns and stereographic projections along zone axes are included) ($\overrightarrow{b_1}$ to $\overrightarrow{b_4}$: ⟨111⟩ directions; $\overrightarrow{b_5}$: ⟨100⟩ directions) (arrow: dislocation slip direction; numbers: dislocation counts for 1/2⟨111⟩ and ⟨001⟩ Burgers vector).

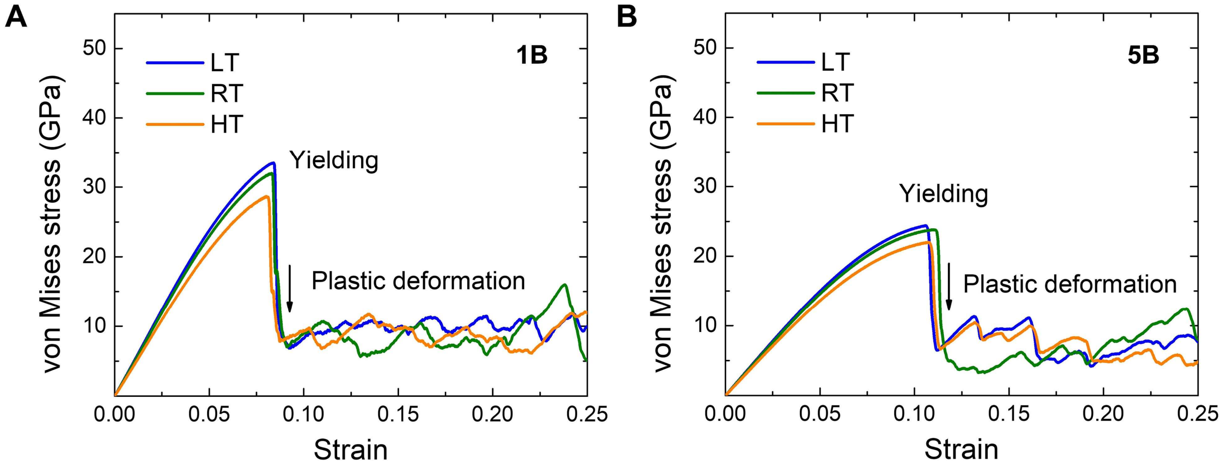

Figure S11. Simulated stress–strain curves along ⟨100⟩ stress orientation at different temperatures: (A) 1B, and (B) 5B.

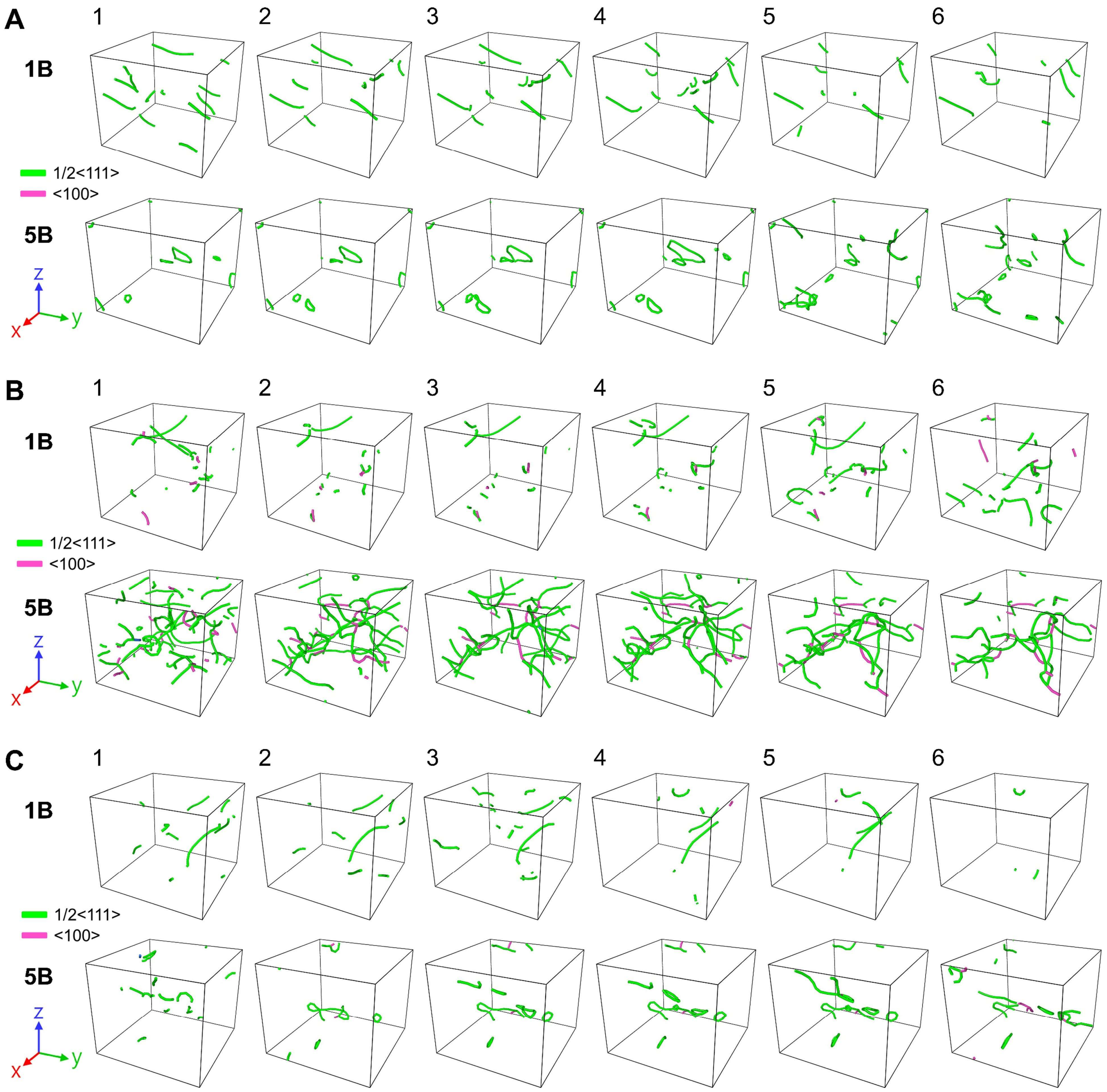

Figure S12. Dislocation evolution for 1B and 5B along ⟨100⟩ stress orientation during the early stage of plastic deformation at different temperatures: (A) LT, (B) RT, and (C) HT (1st stage strain: 9.06% for 1B_LT, 11.50% for 5B_LT, 9.10% for 1B_RT, 11.50% for 5B_RT, 9.50% for 1B_HT, and 11.00% for 5B_HT; 0.5% strain between stages).

Supplementary Videos

Video S1. Video of *in-situ* SEM observation on the microcompression of 1B ⟨100⟩ micropillar #1 deformed at RT.

Video S2. Video of *in-situ* SEM observation on the microcompression of 1B ⟨100⟩ micropillar #2 deformed at RT.

Video S3. Video of *in-situ* SEM observation on the microcompression of 1B ⟨100⟩ micropillar #3 deformed at RT.

Video S4. Video of *in-situ* SEM observation on the microcompression of 1B ⟨100⟩ micropillar #4 deformed at RT.

Video S5. Video of *in-situ* SEM observation on the microcompression of 3B ⟨100⟩ micropillar #1 deformed at RT.

Video S6. Video of *in-situ* SEM observation on the microcompression of 3B ⟨100⟩ micropillar #2 deformed at RT.

Video S7. Video of *in-situ* SEM observation on the microcompression of 3B ⟨100⟩ micropillar #3 deformed at RT.

Video S8. Video of *in-situ* SEM observation on the microcompression of 3B ⟨100⟩ micropillar #4 deformed at RT.

Video S9. Video of *in-situ* SEM observation on the microcompression of 5B ⟨100⟩ micropillar #1 deformed at RT.

Video S10. Video of *in-situ* SEM observation on the microcompression of 5B ⟨100⟩ micropillar #2 deformed at RT.

Video S11. Video of *in-situ* SEM observation on the microcompression of 5B ⟨100⟩ micropillar #3 deformed at RT.

Video S12. Video of *in-situ* SEM observation on the microcompression of 5B ⟨100⟩ micropillar #4 deformed at RT.

References

(1) Hÿtch, M.; Snoeck, E.; Kilaas, R. Quantitative measurement of displacement and strain fields from HREM micrographs. *Ultramicroscopy* **1998**, *74* (3), 131-146.

(2) Thompson, A. P.; Aktulga, H. M.; Berger, R.; Bolintineanu, D. S.; Brown, W. M.; Crozier, P. S.; in't Veld, P. J.; Kohlmeyer, A.; Moore, S. G.; Nguyen, T. D. LAMMPS-a flexible simulation tool for particle-based materials modeling at the atomic, meso, and continuum scales. *Computer Physics Communications* **2022**, *271*, 108171.

(3) Hiremath, P.; Melin, S.; Bitzek, E.; Olsson, P. A. Effects of interatomic potential on fracture behaviour in single-and bicrystalline tungsten. *Computational Materials Science* **2022**, *207*, 111283.

(4) Byggmästar, J.; Nordlund, K.; Djurabekova, F. Simple machine-learned interatomic potentials for complex alloys. *Physical Review Materials* **2022**, *6* (8), 083801.

(5) Stukowski, A.; Bulatov, V. V.; Arsenlis, A. Automated identification and indexing of dislocations in crystal interfaces. *Modelling and Simulation in Materials Science and Engineering* **2012**, *20* (8), 085007.

(6) Zhang, Y.; Zhou, Y. J.; Lin, J. P.; Chen, G. L.; Liaw, P. K. Solid-solution phase formation rules for multi-component alloys. *Advanced engineering materials* **2008**, *10* (6), 534-538.

(7) Raducanu, D.; Cojocaru, V. D.; Nocivin, A.; Hendea, R. E.; Ivanescu, S.; Stanciu, D.; Trisca-Rusu, C.; Serban, N.; Drob, S. I.; Campian, R. S. Microstructure evolution during mechanical alloying of a biodegradable magnesium alloy. *Crystals* **2022**, *12* (11), 1641.

(8) Senkov, O. N.; Wilks, G. B.; Scott, J. M.; Miracle, D. B. Mechanical properties of Nb25Mo25Ta25W25 and V20Nb20Mo20Ta20W20 refractory high entropy alloys. *Intermetallics* **2011**, *19* (5), 698-706.

(9) Jha, S.; Muskeri, S.; Yang, Y. C.; Sadeghilaridjani, M.; Bhowmick, S.; Mukherjee, S. Small-Scale Deformation Behavior of Refractory High Entropy Alloy as a Function of Strain Rate and Temperature. *Available at SSRN 4001342*.

(10) Senkov, O.; Scott, J.; Senkova, S.; Meisenkothen, F.; Miracle, D.; Woodward, C. Microstructure and elevated temperature properties of a refractory TaNbHfZrTi alloy. *Journal of Materials Science* **2012**, *47* (9), 4062-4074.

(11) Juan, C.-C.; Tsai, M.-H.; Tsai, C.-W.; Lin, C.-M.; Wang, W.-R.; Yang, C.-C.; Chen, S.-K.; Lin, S.-J.; Yeh, J.-W. Enhanced mechanical properties of HfMoTaTiZr and HfMoNbTaTiZr

refractory high-entropy alloys. *Intermetallics* **2015**, *62* (76), e83.

(12) Senkov, O.; Senkova, S.; Miracle, D.; Woodward, C. Mechanical properties of low-density, refractory multi-principal element alloys of the Cr–Nb–Ti–V–Zr system. *Materials Science and Engineering: A* **2013**, *565*, 51-62.

(13) Senkov, O.; Woodward, C.; Miracle, D. Microstructure and properties of aluminum-containing refractory high-entropy alloys. *Jom* **2014**, *66* (10), 2030-2042.

(14) Harris, K.; Erickson, G.; Sikkenga, S.; Brentnall, W.; Aurrecoechea, J.; Kubarych, K. Development of two rhenium-containing superalloys for single-crystal blade and directionally solidified vane applications in advanced turbine engines. *Journal of materials engineering and performance* **1993**, *2* (4), 481-487.

(15) Donachie, M. J.; Donachie, S. J. *Superalloys: a technical guide*; ASM international, 2002.

(16) Bhujangrao, T.; Veiga, F.; Suárez, A.; Iriondo, E.; Mata, F. G. High-temperature mechanical properties of IN718 alloy: comparison of additive manufactured and wrought samples. *Crystals* **2020**, *10* (8), 689.
